# AXER is an ATP/ADP exchanger in the membrane of the endoplasmic reticulum

**DOI:** 10.1038/s41467-018-06003-9

**Published:** 2018-08-28

**Authors:** Marie-Christine Klein, Katharina Zimmermann, Stefan Schorr, Martina Landini, Patrick A. W. Klemens, Jacqueline Altensell, Martin Jung, Elmar Krause, Duy Nguyen, Volkhard Helms, Jens Rettig, Claudia Fecher-Trost, Adolfo Cavalié, Markus Hoth, Ivan Bogeski, H. Ekkehard Neuhaus, Richard Zimmermann, Sven Lang, Ilka Haferkamp

**Affiliations:** 1 Medical Biochemistry and Molecular Biology, Saarland University, 66421 Homburg, Germany; 2 Biophysics, CIPMM Saarland University, 66421 Homburg, Germany; 3 Plant Physiology, Technical University Kaiserslautern, 67663 Kaiserslautern, Germany; 4 Physiology, CIPMM Saarland University, 66421 Homburg, Germany; 5 Center for Bioinformatics, Saarland University, 66041 Saarbrücken, Germany; 6 Experimental and Clinical Pharmacology and Toxicology, Saarland University, 66421 Homburg, Germany; 7 Molecular Physiology, University Medical Center, University of Göttingen, 37073 Göttingen, Germany

## Abstract

To fulfill its role in protein biogenesis, the endoplasmic reticulum (ER) depends on the Hsp70-type molecular chaperone BiP, which requires a constant ATP supply. However, the carrier that catalyzes ATP uptake into the ER was unknown. Here, we report that our screen of gene expression datasets for member(s) of the family of solute carriers that are co-expressed with BiP and are ER membrane proteins identifies SLC35B1 as a potential candidate. Heterologous expression of SLC35B1 in *E. coli* reveals that SLC35B1 is highly specific for ATP and ADP and acts in antiport mode. Moreover, depletion of SLC35B1 from HeLa cells reduces ER ATP levels and, as a consequence, BiP activity. Thus, human SLC35B1 may provide ATP to the ER and was named AXER (ATP/ADP exchanger in the ER membrane). Furthermore, we propose an ER to cytosol low energy response regulatory axis (termed lowER) that appears as central for maintaining ER ATP supply.

## Abstract (web summary)

Endoplasmic reticulum (ER) functioning requires a constant supply of ATP, but the exchanger required for ATP uptake into the ER is unknown. Here, the authors report that SLC35B1, here named AXER, or ATP/ADP exchanger into the ER, can transport ATP into the ER.

## Introduction

In order to play its central role in protein biogenesis, the endoplasmic reticulum (ER) of nucleated cells depends on an Hsp70-type molecular chaperone, termed immunoglobulin heavy chain binding protein (BiP, also called glucose-regulated protein, Grp78)^1,2^. BiP is present in the ER lumen in millimolar concentration and requires a constant supply of ATP for its various functions^3–7^. Moreover, ATP hydrolysis by BiP generates ADP and, therefore, necessitates ADP removal from the ER. Although, ER membrane-resident ATP/ADP antiporters have been described for the plant *Arabidopsis thaliana* (ER-ANT1) and for the alga *Phaeodactylum tricornutum* (*Pt*NTT5)^8,9^, these proteins do not represent the major carriers for chemical energy in the ER of these organisms. Thus, although ATP entry into the ER is strictly required for cell function, ubiquitous proteins catalyzing the corresponding ATP uptake, and the concomitant ADP release remained unknown on the molecular level.

Screening databases for solute carriers (SLCs)^10,11^ that are located in the ER membrane (GeneCards: http://www.genecards.org, The Human Protein Atlas: https://www.proteinatlas.org) and that show the same expression pattern in human tissues as BiP, which is the main ATP-consumer in the ER lumen (GenesLikeMe: https://genecards.weizmann.ac.il/v3/index.php?path=/GenesLikeMe), focussed our attention on SLC35B1 (UniProtKB/Swiss-Prot: P78383.1). Human SLC35B1 has up to three different isoforms that are encoded by different mRNA variants. SLC35B1 is a member of the nucleotide-sugar transporter family^12–14^, hence its synonym UDP-galactose transporter-related protein 1 (UGTrel1), and orthologs are present in diverse eukaryotes. SLC35B1 is predicted to have ten transmembrane helices and to be structurally related to members of the drug/metabolite transporter (DMT) superfamily, which also includes the amino acid transporter *Sn*YddG, the structure of which was recently solved^10,11,15^.

Here, we show that heterologously expressed SLC35B1 is highly specific for ATP and ADP and operates in antiport mode, two of four characteristics it shares with the ATP transport activity which is present in rough ER membranes. Moreover, depletion of SLC35B1 from HeLa cells reduces ER ATP levels and, therefore, BiP activity, which implies that SLC35B1 mediates ATP uptake into the ER plus ADP release from the ER in vivo. Thus SLC35B1 operates as an ATP/ADP exchanger in the ER membrane. Furthermore, we initially characterize a regulatory circuit that appears to maintain the ATP supply in the ER and is termed ER low energy response or lowER.

## Results

### SLC35B1 is an ER membrane protein in HeLa cells

**Fig. 1:**
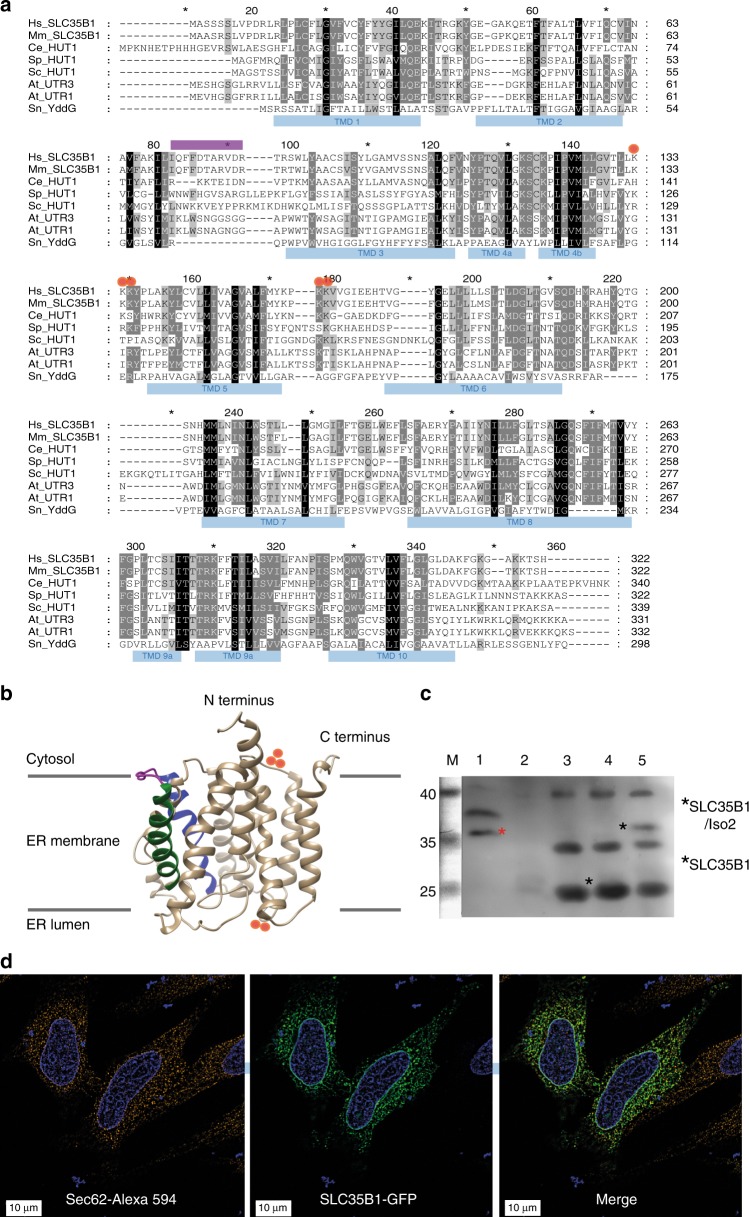
Putative structure and intracellular localization of SLC35B1. **a** Protein sequences are from UniProt or GeneBank and shown in single letter code for *Homo sapiens* (Hs, P78383.1; NM_005827.1), *Mus musculus* (Mm, P97858.1), *Caenorhabditis elegans* (Ce, CAC35849), *Schizosaccharomyces pombe* (Sp, CAB46704.1), *Saccharomyces cerevisiae* (Sc, CAA97965), *Arabidopsis thaliana* (At, At1g14360 and At2g02810), and *Starkeya novella* (YddG, gi:502932551). The sequences were aligned using ClustalX and GeneDoc. The amino and carboxy termini face the cyosol, the double lysine motif near the carboxy terminus of mammalian SLC35B1 serves as ER retention motif. The predicted IQ motif, unique to mammalian SLC35B1, is shown in purple, positively charged clusters in red. SLC35B1/Isoform 2 comprises an amino-terminal extension of 37 amino acids (MRPLPPVGDVRLWTSPPPPLLPVPVVSGSPVGSSGRL) (NM_005827.2), in transcript variant 2 (NM_001278784.1) the first 78 amino acids, including two N-terminal transmembrane helices, of SLC35B1 are replaced by the oligopeptide: MCDQCCVCQDL. **b** Hypothetical structural model of human SLC35B1, as predicted by the Phyre2 server^34^. Transmembrane helices 2 (green) plus 3 (blue) and the connecting loop (purple) with the putative IQ motif are highlighted, as are clusters of positively charged amino acid residues (red). **c** A 4% digitonin extract of canine pancreatic rough microsomal membrane proteins (derived from 6 mg microsomal protein) was subjected to SDS-PAGE in parallel to *E. coli* membranes (25 µg protein), which were derived from non-transfected and SLC35B1-expressing or SLC35B1/isoform 2-expressing cells. The Western blot was decorated with SLC35B1-specific antibody, validated in Supplementary Fig. 1a, and visualized with peroxidase-coupled secondary antibodies, Super Signal West Pico, and luminescence imaging. Molecular mass standard (M) was run in parallel and electronically copied from the stained blot to the Western blot. The relevant part of the blot is shown; the complete blot is shown in Supplementary Fig. 1b. **d** HeLa cells were transfected with an expression plasmid encoding SLC35B1-GFP for 8 h, the nuclei were stained with DAPI, and the ER was visualized with Sec62-specific antibody plus Alexa-Fluor-594-coupled secondary antibody and subjected to fluorescence imaging using a super-resolution Elyra microscope^38^. Representative images and merged images are shown (scale bar 10 µm). Related Western blots are shwon in Supplementary Fig. 1c, d

Human SLC35B1 comes in three different isoforms (Fig. 1a) and is predicted to have ten transmembrane helices (Fig. 1b). First, we confirmed the ER localization of SLC35B1 by demonstrating the presence of SLC35B1/Isoform 2 (NM_005827.2) by immunoblot with a specific anti-SLC35B1 antibody (Fig. 1c, red asterisk in lane 1, Supplementary Fig. 1a, b) in a highly enriched membrane protein extract from pancreatic rough microsomes, which are routinely used for the analysis of ER protein import^6,7^ and as a source of mammalian ER proteins^16^. Notably, the amount of extract that was needed to allow SLC35B1 detection by Western blot corresponded to 6 mg of total microsomal protein, suggesting that SLC35B1 is not an abundant protein in canine pancreas. Therefore, it was not technically feasible to address the question of which isoform(s) is (are) present in HeLa cells, which is consistent with the low reported level of native SLC35B1 in HeLa cells (15 nM^17^; Supplementary Table 1).

Next, we expressed GFP-tagged SLC35B1 in HeLa cells at a moderate level (Supplementary Fig. 1c, d) and confirmed its ER localization by colocalization with the ER protein Sec62 using super-resolution microscopy (Fig. 1d). Heterologous expression in *E. coli* confirmed that the GFP-tag did not affect carrier activity (see below). As a caveat, we admit that it would have been desireable to have a second confirmation for ER localization of SLC35B1 in HeLa cells, e.g., by immunofluorescence microscopy after knocking in an antibody-targetable variant into the endogenous locus. However, we refrained form using this strategy under the assumption that the used strategy of transient expression allowed for finding a better compromise between expression level and detection sensitivity.

Furthermore, we expressed Myc-DDK-tagged SLC35B1/Isoform 2 in HeLa cells at a moderate level (Supplementary Fig. 1e, f) and employed immunoprecipitation in combination with subsequent mass spectrometry to address the question in which cellular compartments potential interaction partners are located. Mock-transfected cells served as negative control. SLC35B1 was efficiently immunoprecipitated from detergent solubilized HeLa cells with ANTI-FLAG M2 affinity gel and not found in the negative control immunoprecipitation (Supplementary Table 2, position 41). Among the 50 co-immunoprecipitated proteins with the highest total peptide scores we detected 26 proteins of the ER or ER-derived vesicles, 10 plasma membrane proteins, 7 mitochondrial proteins, 3 proteins of the Golgi, 2 endosomal proteins, 1 protein of the inner nuclear membrane, and 1 ribosomal protein (Supplementary Table 2). Notably, 19 of these co-immmunoprecipitated proteins were previously found to be co-immunoprecipitated with a bona fide ER protein (hSND2/TMEM208)^18^, including 4 plasma membrane and 2 mitochondrial proteins. Thus, the SLC35B1 interactome also supports the conclusion that SLC35B1 is a protein of the human ER membrane. Its predominant interaction partners are major players in ER protein import (BiP, Calnexin, Oligosaccharyltransferase, Sec61 complex, TRAP complex) and/or cellular calcium homeostasis (ITPR1 and 3, SERCA2). These results are consistent with the localization of human SLC35B1 according to The Human Protein Atlas (https://www.proteinatlas.org) and with the localization of SLC35B1 in *C. elegans*^13^, *S. cerevisiae*^12^, *S. pombe*^12^, and *A. thaliana* (AtUTr1)^14^.

### Heterologously expressed SLC35B1 is an ATP/ADP antiporter

**Fig. 2:**
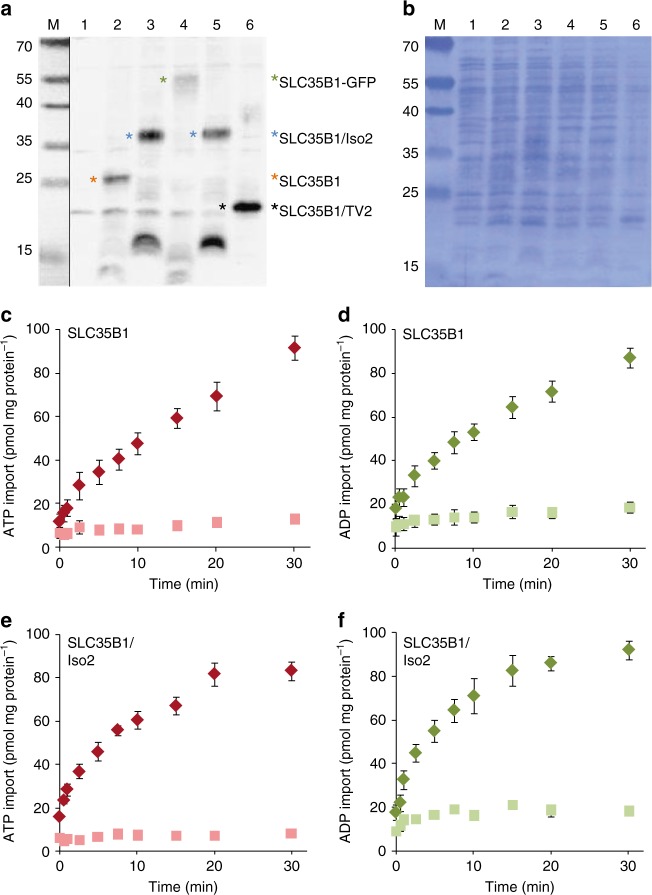
Heterologously expressed SLC35B1 and SLC35B1/Isoform 2 are ATP and ADP carriers. The indicated SLC35B1 variants (labeled by asterisk in **a**, which were described in the legend to Fig. 1a, plus a phosphomimetic variant of Isoform 2 with aspartate residues at positions 15 and 29 instead of serines (termed Isoform 2DD, lane 5), and the GFP-tagged version of SLC35B1 were expressed in different *E. coli* cells. **a**, **b** Membranes were isolated from the indicated *E. coli* cells, and samples (25 µg protein) analyzed by SDS-PAGE and Western blotting using an SLC35B1-specific antibody, which was characterized in Supplementary Fig. 1a (**a**). Non-induced cells served as a negative control and are shown in lane 1. Coomassie staining the blot for total protein served as a loading control (**b**). A molecular mass standard (M) was run in parallel and electronically copied from the stained blot to the Western blot image. **c**–**f** Uptake of 50 µM [α^32^P]ATP (**c**–**e**) or [α^32^P]ADP (**d**–**f**) into SLC35B1-expressing (**c**, **d**) or SLC35B1/Isoform 2-expressing cells (**e**, **f**) was measured and compared to uptake by non-induced cells. Data are reported as the mean of at least three independent experiments and are shown with the standard error of the mean (SEM). Related substrate saturation experiments and corresponding Eadie-Hofstee analyses revealed apparent K_M_ values for ATP and ADP plus maximal uptake rates and are shown in Supplementary Fig. 4

To test whether SLC35B1 might act as an ATP/ADP transporter, we expressed cDNAs for SLC35B1 (P78383.1, NM_005827.1) and SLC35B1/Isoform 2 (NM_005827.2) in *E. coli* cells, routinely used to characterize nucleotide transport proteins^8,9,19^. First of all, we investigated whether the recombinant proteins are integrated into the plasma membrane using Western blot analysis. Indeed, the two SLC35B1 isoforms were expressed and inserted into the bacterial membrane (Fig. 2a, b). Moreover, *E. coli* cells gained ATP and ADP import capacity due to the expression of these carriers (Fig. 2c–f). Non-induced control cells showed neither protein bands that reacted with the anti-SLC35B1 antibody in the membrane fractions (Fig. 2a), nor any substantial accumulation of labeled adenine nucleotides (Fig. 2c–f). The two heterologously expressed SLC35B1 isoforms are highly specific for ATP and ADP, with no competition from AMP, CTP, GTP, UTP, UDP-glucose, or UDP-galactose (its putative substrate) with [α^32^P]ATP import (Table 1).

**Table 1:** Impact of potential substrates and effectors on ATP transport by SLC35B1

Effector	SLC35B1		SLC35B1/1isoform 2	
	Transport (%)	± SEM (%)	Transport (%)	± SEM (%)
ATP	**37.1**	4.5	**31.2**	3.0
ADP	**48.6**	3.1	**43.5**	3.7
AMP	85.1	7.4	88.0	4.3
UTP	114.0	12.6	106.4	12.9
GTP	124.2	16.5	100.1	11.9
CTP	122.6	10.2	109.2	7.7
UDP glucose	129.5	13.5	105.9	8.4
UDP galactose	107.3	5.6	93.9	1.5
UMP	105.4	14.6	98.1	3.9
UDP	126.8	19.5	92.7	2.9
UDP-Nac-Glc-AmN	120.5	13.9	106.5	12.3
GDP mannose	110.4	3.7	99.5	7.9
Mg^2+^ 500 µM	113.4	9.6	106.5	5.2
Ca^2+^ 10 µM	105.5	7.6	106.7	12.5
Ca^2+^ 50 µM	119.8	19.0	109.6	7.0
EDTA 200 µM	146.8	10.8	114.0	10.8
EGTA 20 µM	114.6	4.9	103.6	6.2
EGTA 200 µM	120.8	9.2	105.3	8.6

*Note*: Cellular uptake of 50 µM of [α^32^P]ATP by recombinant SLC35B1 isoforms proceeded for 5 min (considered 100%). The corresponding transport in the presence of selected potential non-labeled substrates (500 µM) or effectors was calculated accordingly. Rates are reported as the mean of at least three independent experiments and represent net values (minus ATP import by non-induced cells). Standard errors of the mean are given (±SEM). Bold values indicate significantly reduced transport (<60% of control)

**Fig. 3:**
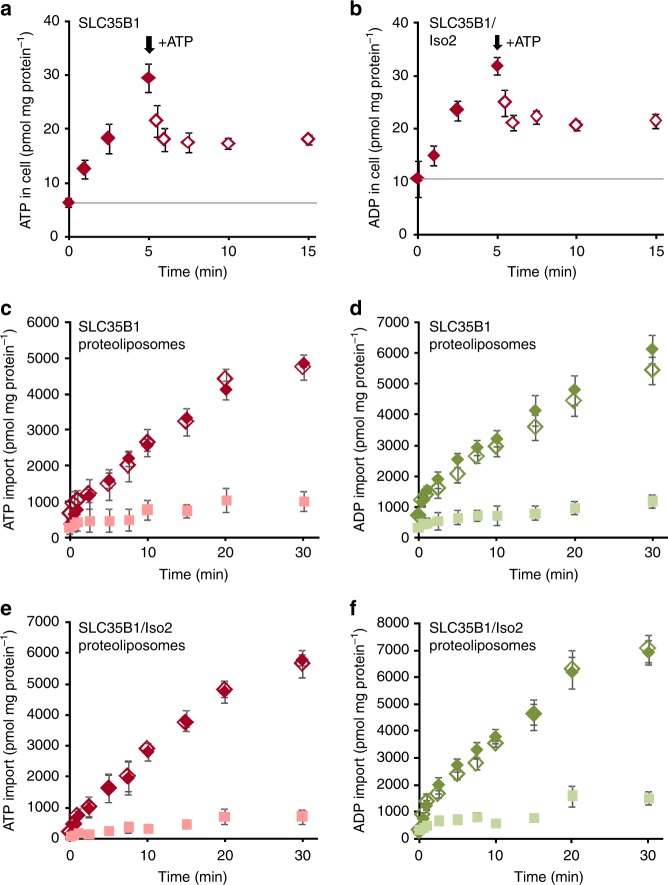
Heterologously expressed SLC35B1 and SLC35B1/Isoform 2 are antiporters for ADP and ATP. **a**, **b** After 5 min of uptake of 20 µM [α^32^P]ATP as described in Fig. 2, efflux was induced by the addition of ten-fold molar excess of unlabeled ATP. Data are reported as the mean of at least three independent experiments and are shown with the standard error of the mean (SEM). See Supplementary Fig. 2 for the demonstration of ATP export into the cell supernatants under similar conditions. **c**–**f** Proteoliposomes harboring membrane proteins from SLC35B1 (**c**, **d**) or SLC35B1/Isoform 2 (**e**, **f**) transfected *E. coli* cells were prepared as described in Methods. Time dependent accumulation of 50 µM [α^32^P]-ATP in unloaded (pink) or preloaded proteoliposomes (**c**, **e**) or 50 µM [α^32^P]-ADP in unloaded (light green) or preloaded proteoliposomes (**d**, **f**). Proteoliposomes were preloaded with 10 mM ADP (empty rhombs) or 10 mM ATP (filled rhombs). [α^32^P]-ATP or [α^32^P]-ADP was present at a concentration of 50 µM. Uptake was terminated after 2 min. Data are reported as the mean of at least three independent experiments and are shown with the standard error of the mean (SEM)

The fact that newly imported [α^32^P]ATP could be chased from the cells by addition of an excess of unlabeled ATP already suggested that both SLC35B1 isoforms act in an antiport mode^9^ (Fig. 3a, b, Supplementary Fig. 2). To further substantiate this transport mechanism, membrane proteins of the respective transfected cells were solubilized in detergent and reconstituted into liposomes. Both, SLC35B1 and SLC35B1/Isoform 2 were able to facilitate the import of ATP or ADP into proteoliposomes, loaded with ADP or ATP (Fig. 3c–f), whereas no import was detectable in absence of loading substrates. This fact verified that the two SLC35B1 isoforms catalyze the strict antiport of ATP and ADP.

A significantly shortened variant of SLC35B1, which was reported to be encoded by transcript variant 2 (NM_001278784.1) and lacks the first two transmembrane domains (including a cluster of six aromatic amino acid residues), however, showed much lower ATP and ADP transport rates than SLC35B1 and SLC35B1/Isoform 2 (Supplementary Fig. 3a, b), although it was present in the *E. coli* membranes at similar concentrations as SLC35B1 (Fig. 2a, b). On the other hand, a heterologously expressed phosphomimetic variant of SLC35B1/Isoform 2 exhibited nearly the same biochemical characteristics as SLC35B1 and SLC35B1/Isoform 2 (Supplementary Fig. 3c, d), as did GFP-tagged SLC35B1 (Supplementary Fig. 3e, f), which was used for SLC35B1 visualization in HeLa cells (Fig. 1d).

### SLC35B1 is similar to the ER resident ATP carrier

**Fig. 4:**
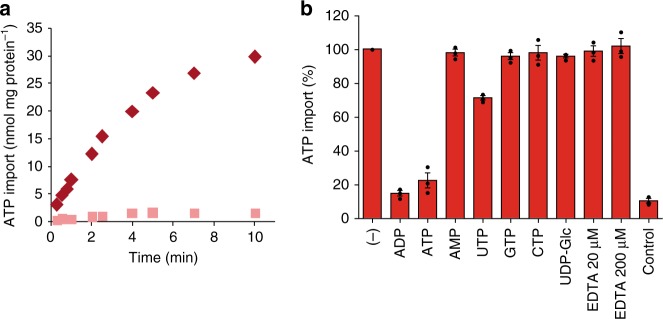
ATP transport into proteoliposomes with the full complement of mammalian ER membrane proteins. Proteoliposomes harboring porcine or canine pancreatic rough ER membrane proteins were prepared as described in Methods. **a** Time dependent accumulation of 50 µM [α^32^P]-ATP in unloaded (pink) or preloaded (10 mM ADP; red) proteoliposomes, which were derived from porcine pancreatic microsomes by treaytment with puromycin and high salt (PKRM). Related substrate saturation experiments and corresponding Eadie–Hofstee analyses are shown in Supplementary Fig. 5. All data are reported as the mean of at least three independent experiments. **b** Proteoliposomes were preloaded with 10 mM ADP (except for the bar labeled control, which refers to unloaded proteoliposomes). [α^32^P]-ATP was present at a concentration of 50 µM. Each effector substance was given at a concentration of 500 µM and uptake was terminated after 2 min. All data are reported as the mean of at least three independent experiments and are shown with the standard error of the mean (SEM)

Heterologously expressed SLC35B1 exhibited similar apparent *K*_M_ and *V*_max_ values for ATP (32.6–34.7 µM and 871.0–904.5 pmol mg protein^−1^ h^−1^; correlation: 97.5%) and ADP (32.0–37.3 µM and 888.4–962.3 pmol mg protein^−1^ h^−1^; correlation: 95.7%) (Supplementary Fig. 4a–d), as did SLC35B1/Isoform 2 (Supplementary Fig. 4e–h). In each case, the two numbers were derived from substrate saturation experiments and corresponding Eadie-Hofstee analyses. These apparent affinities of SLC35B1 for ATP are in line with data on ATP and ADP import into proteoliposomes, harboring the full complement of yeast^20^ or mammalian ER membrane proteins (Fig. 4a, Supplementary Fig. 5). In none of these eukaryotic systems, however, did the biochemical approach identify the carrier^21–23^. Notably, substrate specificity and antiport mode of the two SLC35B1 isoforms were also shared by the ATP/ADP carrier, which were present in proteoliposomes, harboring the full complement of mammalian ER membrane proteins (Fig. 4). ATP import into these proteoliposomes was dependent on their preloading with ADP (Fig. 4a) and not competed by AMP or other nucleoside triphosphates (Fig. 4b). Furthermore, the two heterologously expressed SLC35B1 isoforms and the ER membrane resident ATP transport activity share an insensitivity towards EDTA, i.e., transport Mg^2+^-free ATP (Table 1, Fig. 4b). As a caveat, we concede that it would have been ideal to additionally get the direct comparison to proteoliposmes with the full complement of ER membrane proteins from SLC35B1 over-producing HeLa cells. However, we refrained from trying this strategy assuming that it is not technically feasible to purify ER membrane proteins from HeLa cells in the required quantity and purity (i.e., without contaminating mitochondria).

Based on the ATP and ADP transport characteristics of the heterologously expressed SLC35B1 isoforms 1 and 2 and their similarities to the ATP and ADP transport activities that are present in proteoliposomes with the full complement of mammalian ER membrane proteins, the two SLC35B1 isoforms 1 and 2 are good candidates to work as ATP/ADP exchangers in the human ER membrane.

### Depletion of SLC35B1 from HeLa cells reduces ER ATP levels

**Fig. 5:**
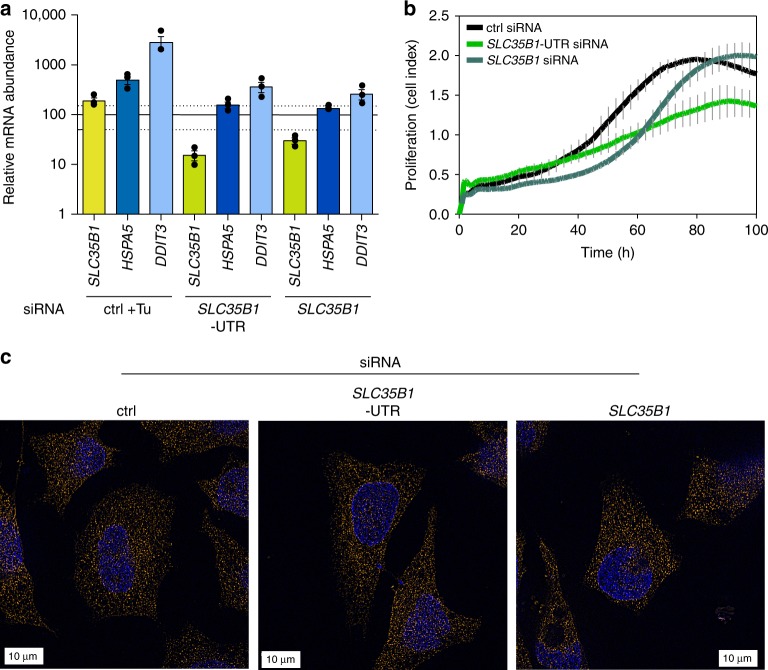
*SLC35B1* knockdown in HeLa cells does not dramatically affect cell growth, cell and ER morphology. **a** Quantitative RT-PCR performed after the transfection of HeLa cells with control siRNA or with *SLC35B1*- or *SLC35B1*-UTR targeting siRNA for 96 h. One set of control siRNA samples was treated with tunicamycin (Tu, 2.5 µg/ml) for 2 h in order to induce UPR^40^. Data from three independent experiments are reported together with the individual data points as % of control with SEM. The light lines indicate the 50 and 150% values. The *HSPA5* gene encodes BiP, the *DDIT3* gene CHOP. **b** HeLa cells were transfected with control siRNA or with *SLC35B1*-targeting or *SLC35B1*-UTR-targeting siRNA for 48 h, transferred to xCELLigence plates (5 × 10^3^ cells per well), and cultivated for 100 h. Cell growth was monitored in real-time. **c** HeLa cells were transfected with control siRNA or with *SLC35B1*-UTR-targeting siRNA for 96 h, stained with DAPI and with a Sec62-specific antibody, and analyzed on the super-resolution microscope^38^. Representative images are shown (scale bar 10 µm)

To directly address whether SLC35B1 acts as a transporter of chemical energy in the ER in human cells, HeLa cells were treated with two different *SLC35B1*-targeting siRNAs for 96 h, and the knockdown efficiencies were evaluated by quantitative RT-PCR (qRT-PCR) analysis. The analysis showed that mRNA depletion was efficient: the 5´ untranslated region (UTR)-targeting siRNA knocked down the residual *SLC35B1* level to ~10% and the coding region-targeting siRNA to ~20% (Fig. 5a). Western blot analysis with the anti-SLC35B1 antibody indicated that the siRNAs cause efficient protein depletion in SLC35B1-GFP expressing cells (Supplementary Fig. 1c). However, the Western blot approach for endogeneous SLC35B1 failed, most likely due to the low level of native SLC35B1 in HeLa cells^17^ (see Supplementary Fig. 1e). Depletion of SLC35B1 for 96 h did not cause any major alterations in whole cell or ER morphology, but led to slight over-expression of the apoptosis inducer CHOP^24^ and to concomitant decelerated cell growth, which was more pronounced for the more efficient *SLC35B1*-UTR siRNA (Fig. 5b, c).

**Fig. 6:**
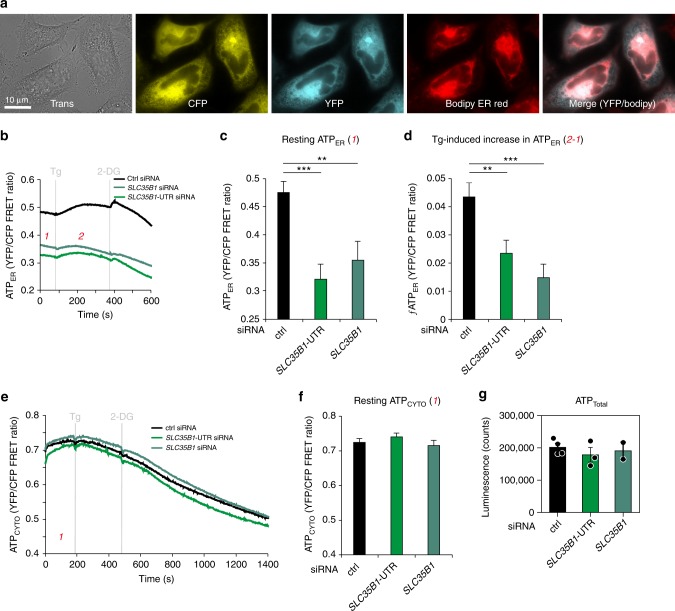
ERAT4.01 reveals ATP depletion in the ER of HeLa cells after *SLC35B1* knockdown. **a**–**d** HeLa cells were transfected with control siRNA or with *SLC35B1*- or *SLC35B1*-UTR-targeting siRNA for 72 h, then transfected with ERAT4.01. After 24 h, they were imaged by fluorescence microscopy. Where indicated, Thapsigargin (Tg, 1 µM) or 2-deoxy-glucose (2-DG, 10 mM) were added. **a** Images were recorded using transmission light or fluorescence. Representative images are shown (scale bar 10 µm). **b** Time-resolved live cell recordings of ER luminal ATP levels are shown as FRET-ratio F_535_/F_480_. Data are presented as means for ctrl, *n* = 33 cells, UTR siRNA, *n* = 20, *SLC35B1* siRNA, *n* = 21, from at least three independent experiments. **c** Statistical analysis of the resting ATP levels in the experiments shown in **b**. Three time points before Tg addition were averaged (indicated as 1) and subtracted from the MAX-values (indicated as 2) following Tg addition for each single cell. Data are presented as mean with SEM. The indicated pairs were assessed by unpaired, two-sided standard Student´s *t*-test (*P *<* 0.05, **P *<* 0.01, **P *<* 0.001). **d** Statistical analysis of the Tg-induced ATP increase in the experiments shown in **b**. **e**, **f** HeLa cells were transfected with control siRNA or with *SLC35B1*- or *SLC35B1*-UTR-targeting siRNA, transfected with ATeam, and imaged. The mean values of time-resolved live cell recordings of cytosolic ATP levels (**e**) and the corresponding statistical analysis (**f**) are shown for ctrl, *n* = 116 cells, UTR siRNA, *n* = 95, *SLC35B1* siRNA, *n* = 57 from at least three independent experiments. **g** HeLa cells were transfected with control siRNA or with *SLC35B1*-targeting or *SLC35B1*-UTR-targeting siRNA for 96 h, and total cellular ATP was determined using ApoSensor according to the manufacturer´s protocol. Data from three independent experiments are reported together with the individual data points as % of control with SEM

**Fig. 7:**
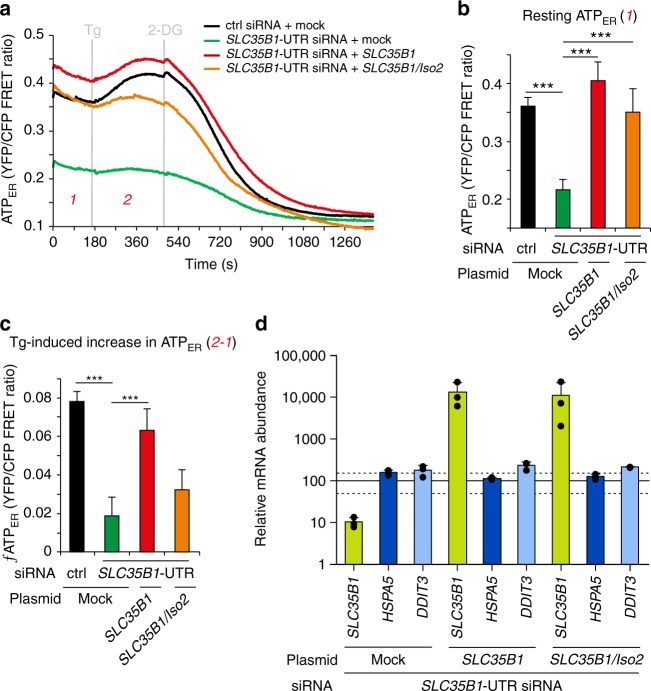
*SLC35B1* expression rescues ATP levels in the ER after *SLC35B1* knockdown. **a**–**c** HeLa cells were transfected with control siRNA or with *SLC35B1*-UTR-targeting siRNA plus *SLC35B1* expression plasmids for 72 h. The cells were transfected with ERAT4.01. After 24 h, they were imaged by fluorescence microscopy, and the FRET-ratio F_535_/F_480_ was recorded in real-time. Where indicated, 1 µM Tg or 10 mM 2-DG were added. **a** Time-resolved live cell recordings of ER luminal ATP levels. Data are presented as means of ctrl, *n* = 71 cells, UTR siRNA, *n* = 39, SLC rescue, n = 18, and Iso2 rescue, n = 13 from at least three independent experiments. **b** Quantification of resting ATP ER concentration in **a**. **c** Quantification of Tg-induced ATP ER concentration in **a**. Three time points before Tg addition were averaged (indicated as 1) and subtracted from the MAX-values (indicated as 2) following Tg addition for each single cell. Data in **b** and **c** are presented as mean with SEM. The indicated pairs were assessed by unpaired, two-sided standard Student´s *t*-test (*P *<* 0.05, **P *<* 0.01, **P *<* 0.001). **d** Mean quantitative RT-PCR values are shown after transfection of HeLa cells with control siRNA or with *SLC35B1*-UTR targeting siRNA plus *SLC35B1* expression plasmids. Data from three independent experiments are reported together with the individual data points as % of control with SEM. The light lines indicate the 50 and 150% values. The *HSPA5* gene encodes BiP, the *DDIT3* gene CHOP

Next, the energy status of SLC35B1-depleted cells was characterized with time-resolved live cell recordings of ER ATP levels using the ER-targeted, genetically encoded ATP FRET sensor ERAT4.01^25^ (Fig. 6a). ATP levels were detected and compared in the ER of HeLa cells treated either with two different *SLC35B1*-targeting or control siRNAs, followed by ERAT4.01 transfection. *SLC35B1* knockdown was correlated with significantly lower ATP levels in the ER compared to control cells (Fig. 6b, phase 1; quantification in Fig. 6c). Thapsigargin (Tg), which inhibits sarcoplasmic/endoplasmic reticulum Ca^2+^ ATPase (SERCA) in the ER membrane and stimulates Ca^2+^ release from the ER, led to the expected increase in the ER ATP levels^25^ (Fig. 6b, phase 2; quantification in Fig. 6d). The succeeding application of 2-deoxy-glucose (2-DG), which reduces the availability of ATP in the cytosol due to inhibition of glycolysis, induced the expected drop in ER ATP levels^25^ (Figs. 6b and 7a). In contrast, the response of *SLC35B1* knockdown cells to both Tg and 2-DG was less pronounced (Figs. 6b, d and 7a). Thus, *SLC35B1* knockdown in HeLa cells reduced ATP levels in the ER and these levels could not be replenished by Tg-induced Ca^2+^ efflux from the ER.

To further substantiate the observed siRNA effects, we performed complementation analyses. Specifically, the ATP imaging experiments that were conducted in the presence of the *SLC35B1*-UTR-targeting siRNA were repeated in the presence of *SLC35B1*-expression or *SLC35B1*/Isoform 2-expression plasmids that lacked the UTR. Under these complementation conditions, the ATP levels in the ER were rescued to the levels in the control conditions, and the rescued cells responded to Tg and 2-DG with the expected increase and decrease, respectively, in ATP levels in the ER, similar to the control cells (Fig. 7a–d).

In summary, the results from the heterologous expression experiments, *SLC35B1* knockdown in HeLa cells in combination with live cell imaging of ATP levels in the ER, and complementation experiments indicated that SLC35B1 and SLC35B1/Isoform 2 represent the ATP/ADP exchangers in the human ER membrane that are responsible for net import of chemical energy into the ER.

### Depletion of SLC35B1 from HeLa cells inhibits BiP activity

**Fig. 8:**
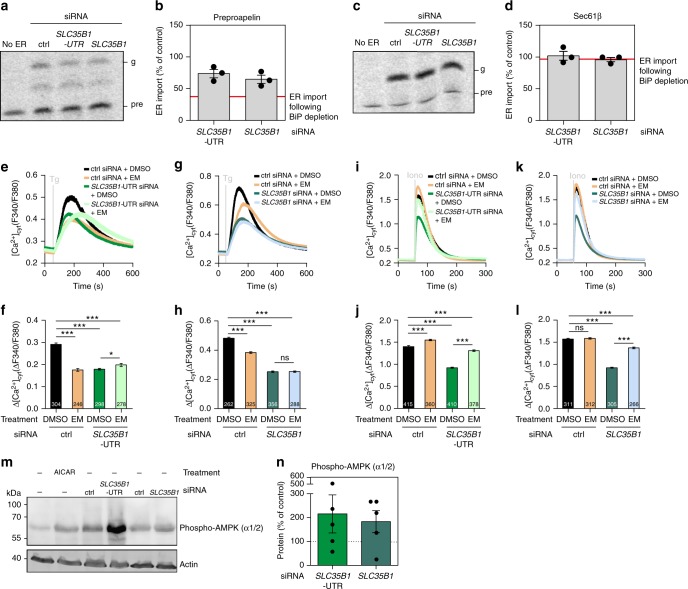
Knockdown of *SLC35B1* leads to inhibition of BiP activity and to AMPK phosphorylation. **a**–**d** HeLa cells were transfected with control siRNA or with *SLC35B1*-targeting or *SLC35B1*-UTR-targeting siRNA for 96 h. They were converted to semi-permeabilized cells and tested for protein transport activity as described previously^6,26,38,39^. Here, ER protein import, assayed as N-glycosylation, of the small presecretory protein preproapelin^26^ (which depends on BiP, (**a**, **b**), and of the tail anchored Sec61ß^26^ (which does not depend on BiP, (**c**, **d**) were analyzed. The effects of siRNA-mediated depletion of BiP are shown for comparison^26^. Representative phosphorimages after SDS-PAGE and quantitative data from three independent experiments are reported together with the individual data points as % of control with SEM. Pre, precursor form; g, glycosylated form. **e**–**l** HeLa cells were transfected with control siRNA or with *SLC35B1*- or *SLC35B1*-UTR-targeting siRNA for 96 h. Where indicated, 0.0001% DMSO, or 10 µM emetine (EM) were present during the last 2 h of growth plus Fura loading. Cells were loaded with Fura-2 for 30 min, transferred to Ca^2+^-free buffer, and Fura-2 signals were recorded as F_340_/F_380_ ratios in real-time^6,27^. Where indicated, 1 µM Thapsigargin (Tg), or 5 µM Ionomycin (Iono) were added. **e**, **g**, **i**, **k** The mean values of the ratiometric recordings are shown with the standard error of the mean (SEM). **f**, **h**, **j**, **l** Statistical analysis of Tg-induced (**f**–**h**) or Iono-induced (**j**–**l**) changes in cytosolic Ca^2+^ levels in the experiments with the indicated number of cells in at least three independent experiments. The indicated pairs were assessed by unpaired, two-sided Student´s *t*-test (*P *<* 0.05, **P *<* 0.01, **P *<* 0.001, ns, not significant). **m**, **n** Where indicated, 0.5 mM AICAR was present for 15 h. Cells were treated as indicated and analyzed by SDS-PAGE and Western blotting using phosphorylated AMPK-specific antibodies. Notably, AICAR is an AMP-mimetic and AMPK activator^46^. Staining the blot for ß-actin served as a loading control. Representative luminescence images of the blots and quantitative data from five independent experiments are reported together with the individual data points as % of control with SEM

ER lumenal ATP allows BiP to fulfill its physiological roles, e.g., in facilitating selective protein import into the ER^6,7,26^ and in limiting Ca^2+^ leakage from the ER^6,16^, both by affecting gating of the Sec61 channel. Therefore, we expected reduced BiP activity as a result of *SLC35B1* knockdown. This was first tested by analyzing BiP-dependent protein import into the ER^26^. In these experiments, SLC35B1 was depleted from HeLa cells by treatment with two different *SLC35B1*-targeting siRNAs for 96 h. Subsequently, the depleted cells were converted to semi-permeabilized cells and analyzed for their in vitro protein import capacity in parallel to semi-permeabilized cells from non-targeting siRNA-treated cells^26^. For protein import analysis, established BiP-dependent (preproapelin)^26^ and BiP-independent precursor polypeptides (Sec61ß)^26^ were synthesized and radiolabeled in rabbit reticulocyte lysate and import efficiency was evaluated via SDS-PAGE and phosphorimaging. BiP-dependent protein import was found to be reduced by *SLC35B1* knockdown, whereas BiP-independent transport was unaffected (Fig. 8a–d). Thus, SLC35B1 depletion phenocopied the effect of BiP depletion on BiP-dependent ER protein import^26^.

In a second approach, HeLa cells were treated with the two different *SLC35B1*-targeting siRNAs for 96 h and loaded with the ratiometric Ca^2+^ indicator Fura-2^6,27^, to address by live cell Ca^2+^ imaging if SLC35B1 depletion also affects BiP´s role in limiting Ca^2+^ leakage from the ER^6,16^. *SLC35B1* knockdown by both siRNAs decreased the Ca^2+^ concentration in the ER lumen, which was measured as Tg-releasable Ca^2+^ (Fig. 8e–g; statistics in Fig. 8f–h), and it also decreased total Ionomycin-releasable Ca^2+^ (Fig. 8i–k; statistics in Fig. 8j–l). Thus, ATP depletion in the ER driven by *SLC35B1* knockdown decreased ER Ca^2+^, i.e., stimulated ER Ca^2+^ efflux, as it had previously been observed after BiP depletion^6^.

Two possibilities could in principle account for this result, decreased Ca^2+^ uptake or increased Ca^2+^ release from the ER. Considering the importance of the Sec61 protein translocon as a passive ER Ca^2+^ release channel^16,27,28^, and the ER luminal chaperone BiP as its allosteric regulator^6,29^, we hypothesized that the observed drop in ATP levels in the ER lumen in response to *SLC35B1* knockdown might activate Ca^2+^ leakage from the ER via open Sec61 channels. This hypothesis was tested by arresting precursor polypeptides in transit through the Sec61 translocon, i.e., blocking the passive Ca^2+^ leak channel, using the elongation inhibitor emetine along with *SLC35B1* knockdown and live cell Ca^2+^ imaging^28^. Emetine rescued Ca^2+^ homeostasis after *SLC35B1* knockdown with both siRNAs by limiting Ca^2+^ efflux from the ER via Sec61 channels (Fig. 8i–k; statistics in Fig. 8j–l).

Thus, depletion of ATP from the ER results in reduced BiP activity, which causes reduced BiP-dependent ER protein import and increased ER Ca^2+^ efflux. Therefore, these results confirm the conclusion that SLC35B1 represents the ATP/ADP exchanger in the human ER membrane.

### SLC35B1 may be part of a Ca^2+^-dependent regulatory circuit

Finally, we tested if SLC35B1 is involved in controling cellular energy homeostasis in response to ER ATP depletion. AMPK is the master regulator of energy metabolism^25,30–32^ and can be activated by a decrease in cytosolic ATP levels or by an increase in cytosolic Ca^2+^ via the calcium/calmodulin dependent kinase kinase 2 (CAMKK2)^31^.

First, we addressed cytosolic ATP levels as the potential AMPK regulator. Therefore, we monitored ATP levels in the cytosol in real-time using the cytosolic ATP sensor ATeam^33^ and in cellular lysates using a bioluminescent assay. SLC35B1 depletion from HeLa cells did not significantly affect cytosolic ATP levels or the total cellular ATP levels (Fig. 6e–g), which is consistent with the facts that HeLa cells are not professional secretory cells and that the ER in HeLa cells does not significantly contribute to the total cellular ATP levels.

**Fig. 9:**
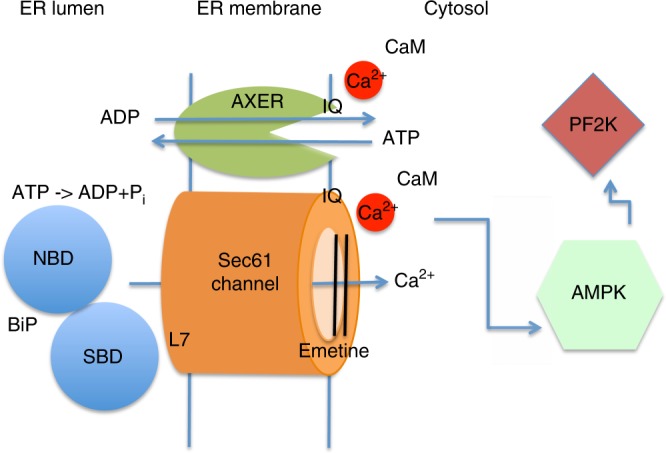
ER low energy response (lowER) ensures a sufficient ATP supply to the human ER. AXER, ATP/ADP exchanger in the ER membrane = SLC35B1; AMPK AMP-activated protein kinase; CaM, Calmodulin; IQ, IQ motif = Ca^2+^-Calmodulin binding site^27^; emetine, inhibitor of polypeptide chain elongation at the ribosome; L7, ER lumenal loop 7 of Sec61α = BiP binding site^6^; NBD, nucleotide binding domain of BiP; PF2K, 6-phospho-fructo-2-kinase; Pi, inorganic phosphate; SBD, substrate binding domain of BiP. Notably, ATP production in HeLa cells relies mainly on glycolysis (in the cytosol) rather than on oxidative phosphorylation (in mitochondria)^25^. See text for details

The results shown in Figs. 6 and 7 already suggested that Tg-induced increase in cytosolic Ca^2+^ and the concomitant increase in ATP levels in the ER were an effect of Ca^2+^ on cytosolic ATP production. Therefore, we hypothesized that the observed drop in ATP levels in the ER lumen in response to *SLC35B1* knockdown might activate a signaling mechanism that controls ER and cytosolic Ca^2+^ levels. This cascade would start with reduction in BiP activity and subsequent increase in Ca^2+^ leakage from the ER via open Sec61 channels. This hypothesis was tested positive in the above-described Ca^2+^ imaging experiments. In brief, *SLC35B1* knockdown decreased the Ca^2+^ concentration in the ER lumen (Fig. 8e–l) and emetine rescued Ca^2+^ homeostasis after *SLC35B1* knockdown by limiting Ca^2+^ efflux from the ER via Sec61 channels (Fig. 8j–l). These results were consistent with previous work that showed that Ca^2+^ efflux from the ER into the cytosol acts as a signal that increases ADP phosphorylation in the cytosol^25^ and expanded the model by adding three additional players, SLC35B1, BiP, and the Sec61 channel (Fig. 9).

At last, we asked how *SLC35B1* knockdown may directly affect cytosolic ATP production. HeLa cells were treated with the two *SLC35B1*-targeting siRNAs for 96 h and the cell lysates were analyzed by Western blotting using antibodies against the phosphorylated alpha and beta subunits of AMPK. We found that *SLC35B1* knockdown resulted in pronounced AMPK phosphorylation (Fig. 8m, n), which is expected to stimulate cytosolic ATP production^25^.

## Discussion

Taking together that SLC35B1 has ATP/ADP exchange acitivity similar to the transport activity that is present in pancreatic ER membranes and may be located in the ER membrane in canine pancreas and human cells, we conclude that SLC35B1 is likely the ATP/ADP exchanger in the ER membrane and suggest to name it AXER. Our findings explain why AXER orthologs in *Saccharomyes cerevisiae* (HUT1), *Schizosaccharomyces pombe* (HUT1), and *Caenorhabditis elegans* (HUT-1) have been found to play a more general role in ER homeostasis, as would be expected for a nucleotide sugar transporter^12,13^.

AXER/Isoform 2 appears to be the only of the three known isoforms present in canine pancreatic microsomes. Which isoform(s) is (are) present in HeLa cells is not completely resolved. We note however that the agarose gel electrophoretic analysis of the PCR products from HeLa cells showed that there are mRNA molecules which are long enough to allow synthesis of Isoforms 1 and 2 (Supplementary Fig. 6), which is consistent with the presence of transcript variants in NCBI. According to UniProtKB serine residues at positions 15 and 29 of AXER/Isoform 2 may be subject to phosphorylation. Therefore, we generated a phosphomimetic variant of AXER/Isoform 2 and characterized its carrier activity after heterologous expression in *E. coli*. We did not detect any effect of the two point mutations under these conditions, i.e., in the absence of potential cytsosolic interaction partners (Supplementary Fig. 3c, d). Thus the phosphorylation per se does not seem to affect carrier activity.

According to a hypothetical structural model of the human AXER (Fig. 1b, Suppplementary Fig. 7a, b), as predicted by the Phyre2 server^34^, AXER can be expected to catalyze the equimolar exchange of adenosine di-phosphates and triphosphates by an alternating access mechanism, in which a single substrate binding site is made available either to the cytosolic ER surface or the ER lumen through conformational changes, which is reminiscent of the mitochondrial ADP/ATP carriers^35^. When AXER is modeled on the X-ray structure of the *Sn*Yddg transporter^15^, two positive clusters that are flanking transmembrane domain 5 on the cytosolic and ER lumenal sides (Fig. 1a, b, Supplementary Fig. 7) suggest another analogy to the mitochondrial carriers, whereby the positive clusters are involved in both steering the substrates to the central cavity and stripping Mg^2+^ from the nucleotides, respectively. Notably, a stripping of Mg^2+^ from the nucleotides is consistent with the observed EDTA insensitivity of both heterologously expressed SLC35B1 and the carrier, which is present in proteoliposomes, harboring the full complement of mammalian ER membrane proteins (Table 1, Fig. 4b). Furthermore, transport direction can be expected to be directed by the ATP gradient.

In human cells, AXER appears to be part of a regulatory circuit and a Ca^2+^-dependent signaling pathway, termed lowER, acting in vicinity of the ER and guaranteeing sufficient ATP supply to the ER (Fig. 9). The initial experimental data to characterize this putative signaling pathway, which were presented here, suggest the following scenario for lowER: High ATP/ADP ratio in the ER allows BiP to limit Ca^2+^ leakage from the ER via the Sec61 channel^6^. Low ATP/ADP ratio due to increased protein import and folding or due to protein misfolding, leads to BiP dissociation from the Sec61 channel thus inducing Ca^2+^ leakage from the ER^6^. In the cytosol, Ca^2+^ binds to calmodulin (CaM) near the ER surface^27^, and activates AMPK via CAMKK2 and finally 6-phospho-fructo-2-kinase (PF2K)^25^. Activated PF2K causes increased ADP phosphorylation in glycolysis, leading to ATP import into the ER via AXER, which is also activated by Ca^2+^ efflux from the ER^25^. Interestingly, mammalian AXER comprises an IQ motif in the cytosolic loop between transmembrane domains 2 and 3 (Fig. 1a, b) and, thus, may also be activated by Ca^2+^-CaM (Supplementary Fig. 8). Normalization of the ER ATP/ADP ratio, causes BiP to limit the Ca^2+^ leakage and thus inactivates the signal transduction pathway. SERCA, which pumps Ca^2+^ back into the ER lumen, balances the passive Ca^2+^ efflux and protein phosphatase 2 (PP2) dephosphorylates AMPK. Note that (i) all mentioned proteins are present in sufficient quantities in the HeLa cells which were used here (Supplementary Table 1); (ii) it is expected that the lowER involves sites of contact between ER and mitochondria and oxidative phosphorylation as an energy source in non-cancer cells; and (iii) activated AMPK was shown previously to lead to reduced cap-dependent translation and therefore ties the lowER to the UPR^30^. While ADP is exported via AXER, phosphate may leave the ER via the Sec61 channel, which is not ion selective^16^ and is also permeable to glutathione^36^.

Our data suggest that AXER is not only an ATP importer and ADP exporter in the ER membrane, but may also be part of an ER to cytosol low energy response regulatory axis (termed lowER). It remains open if AXER is the only ATP carrier in the ER membrane of mammalian cells, its ortholog in *C. elegans* is essential only during larval development but not in the adult worm^13^. The proposed Ca^2+^-dependent regulatory circuit low ER guarantees ER energy metabolism and ER proteostasis under physiological conditions and clearly awaits further validation and characterization. Under non-physiological conditions, it can be expected to represent the first line of defense of a cell against ER stress and, therefore, may interact with the unfolded protein response^30^.

## Methods

### Materials

Expression plasmids for carboxyterminally Myc-DDK-tagged SLC35B1 (RC204145), GFP-tagged SLC35B1 (RG204145), Myc-DDK tagged transcript variant 2 (RC236977), and Myc-DDK-tagged SLC35B1/Isoform2 (RC204145, customized), as well as transient over-expression lysate of SLC35B1 (LY401770) were from OriGene, as was anti-Myc-DDK antibody (TA50011, mouse monoclonal, used dilution: 1:1,000). The expression plasmid for ATP sensor ERAT4.01 was purchased from Next Generation Fluorescence Imaging. ANTI-FLAG M2 affinity gel (A2220), antibodies against β-actin (A5441, mouse monoclonal, used dilution: 1:10,000) and SLC35B1 (HPA057418, rabbit polyclonal, affinity purified, used dilution: 1:200) were obtained from Sigma, antibodies against tGFP (AB514, rabbit polyclonal, used dilution 1:2,500) were from Evrogen, and antibodies against phosphorylated AMPK alpha 1 and 2 (ab133448, rabbit monoclonal, used dilution: 1:1,000) were from ABCAM. Primary antibodies (ß-actin, Myc-DDK) were visualized with ECL^TM^ Plex goat anti-mouse IgG-Cy3 conjugate (Sigma C2181, used dilution: 1:2,500) using the Typhoon-Trio imaging system combined and Image Quant TL software 7.0 (GE Healthcare). Alternatively, they were visualized with peroxidase-coupled secondary antibodies (Sigma A8275, used dilution: 1:1,000), Super Signal West Pico (Pierce) (phosphorylated AMPK) or Super Signal West Femto (Pierce) (SLC35B1), and luminescence imaging using Fusion SL with the FUSION-CAP software 16.11 (PEQLAB). A home-made and affinity purified rabbit antipeptide antibody directed against the COOH terminal undecapeptide of human Sec62 protein (plus an aminoterminal cysteine) and Alexa Fluor594-coupled secondary antibody from goat (Invitrogen A-11012, goat polyclonal, used dilution: 1:1,000) was used for fluorescence microscopy. The ApoSENSOR Bioluminescent Assay Kit was from BioVision and was used according to the manufacturer´s instructions within an infinite M200 plate reader (TECAN). AICAR (5-aminoimidazole-4-carboxamide-1-ß-D-ribofuranoside), and emetine were from Sigma. We note that the full scans of blots and radioactive gels are shown in Supplementary Figs. 9–14.

### Heterologous expression in *E. coli* and nucleotide import

The open reading frames for the respective carrier versions were Gateway cloned into the *E. coli* expression vector pET300 according to the manufacturer’s instructions (Invitrogen). The corresponding primers are listed in Supplementary Table 3. The inserts were confirmed by sequencing. For heterologous expression, Rosetta 2 (DE3)pLysS cells (Novagen) were transformed with the expression vector constructs and grown at 37 °C under aerobic conditions. At an optical density at 600 nm of 0.5, heterologous protein synthesis was induced by the addition of 1 mM isopropyl-β-D-thiogalactopyranosid and the cells were harvested 1 h later by centrifugation (5 min, 5000×*g*, 15 °C). Non-induced cells served as the control. Recombinant protein synthesis and membrane insertion of the recombinant proteins were analyzed by Western blot. First, the pellet was resuspended in 1 mM EDTA, 15% glycerol, 10 mM Tris-HCl, pH 7.0, frozen in liquid nitrogen, and subsequently thawed at 37 °C (5 min). Autolysis was allowed at 37 °C for 5 min, and subsequent sonication on ice was used for cell disruption. Cell debris and protein aggregates were removed by centrifugation (10,000×*g*, 15 min, 4 °C). The membrane proteins in the supernatant were collected by ultracentrifugation (100,000×*g*, 30 min, 4 °C). Proteins were analyzed by SDS–PAGE, Western blotting, and immune detection with SLC35B1-specific antibodies. For analysis of the transport function of the recombinant proteins, the cell pellet was re-suspended in phosphate buffer (50 mM, pH 7.0) directly after harvesting, and 100 µl of the cells (OD_600_ of 5.0) were mixed with 100 µl phosphate buffer supplemented with the indicated final concentrations of [α ^32^P]-labeled ATP or ADP^8^. Transport was conducted at 30 °C and terminated by removal of the external substrate via vacuum filtration and washing (3 × 4 ml phosphate buffer washes). Radioactivity in the cell samples on the filters was quantified by scintillation counting (Tricarb 2500, Canberra-Packard).

### HeLa cell experiments

HeLa cells (DSM no. ACC 57) were obtained from the German Collection of Microorganisms and Cell Cultures, routinely tested for mycoplasma contamination by VenorGeM Mycoplasm Detection Kit (Biochrom AG, WVGM), and replaced every five years by a new batch. They were cultivated at 37 °C in a humidified environment with 5% CO_2_, in DMEM with 10% fetal bovine serum (FBS; Sigma) plus 1% penicillin and streptomycin. Cell growth was monitored using the Countess^®^ Automated Cell Counter (Invitrogen) following the manufacturer’s instructions. Alternatively, cell growth was monitored in real-time in an xCELLigence SP system (Roche Diagnostics) following the manufacturer’s instructions^37^.

For gene silencing, 6.0 × 10^5^ HeLa cells were seeded per 6-cm culture plate, followed by incubation in standard culture conditions. For *SLC35B1* silencing, the cells were transfected with a final concentration of 20 nM targeting siRNA (GGUACCCUGCCAUCAUCUAtt, GAGACUACCUCCACAUCAAtt (UTR)) (Qiagen) or with 20 nM AllStars Negative Control siRNA (Qiagen) using HiPerFect Reagent (Qiagen) following the manufacturer’s instructions. After 24 h, the medium was changed and the cells were transfected a second time. Silencing efficiencies were evaluated by qRT-PCR (see below).

To rescue the phenotype after *SLC35B1* silencing with the corresponding human cDNA, cells were treated with *SLC35B1*-UTR siRNA as described above for 96 h. Eight hours after the second transfection, the siRNA-treated cells were transfected with 4 µg of the *SLC35B1* expression plasmid using Fugene HD (Promega). Complementation was evaluated by qRT-PCR.

### Super resolution microscopy

Cell-morphology and ER-morphology were analyzed by super-resolution fluorescence microscopy on an Elyra SIM (Carl Zeiss MicroImaging)^38^. Cells were seeded on glass cover slips and treated as indicated. After 96 h the glass slides were removed and washed twice with cold PBS. Cells were fixed with 4% Paraformaldehyde for 20 min at 4 °C. Fixed cells were permeabilized and blocked with PSS (PBS + 0.1% Saponin + 10% FCS) for 1 h at room temperature. To improve the antigen accessibility RNAse A (Roche) was added to a final concentration of 50 µg/ml. After washing with PSS, indirect immunofluorescence staining with an affinity purified rabbit antipeptide antibody directed against Sec62 protein and Alexa Fluor594-coupled secondary antibody from goat was performed. Notably, the anti-Sec62 antibody is specific for Sec62 under denaturing, as well as native conditions (i.e., Western blot and fluorescence microscopy-signals were quenched after silencing of the *SEC62* gene)^7,38^. Cells were analyzed by microscopy on an Elyra SIM PS1 (Carl Zeiss-MicroImaging). The microscope was equipped with a Plan-Apochromat Oil DIC lense with ×63 magnification and 1.4 numerical aperture (Carl Zeiss) and an iXon^EM^ + 885 EMCCD camera (Andor Technology). Mounting medium was Roti®-Mount FluorCare DAPI (Carl Roth), the oil was Immersol 518 F (Carl Zeiss).

### Protein transport into semi-permeabilized cells

BiP activity was tested as ER protein import activity^26,38,39^. Precursor polypeptides were synthesized in reticulocyte lysate in the presence of [^35^S]methionine for 16 min at 30 °C. After 5 min of incubation with puromycin (final concentration: 1 mM) at 30 °C, buffer or semi-permeabilized cells, resulting in a final concentration of 12,800 cell equivalents/µl, were added and the incubation was continued for 30 min. The cells had previously been treated with targeting or control siRNA for 96 h. Semi-permeabilized-cells were prepared by digitonin treatment from identical cell numbers, adjusted according to OD_280_ in 2% SDS and, eventually, confirmed by SDS-PAGE and protein staining. All samples were analyzed by SDS–PAGE and phosphorimaging (Typhoon-Trio imaging system). Image Quant TL software 7.0 was used for quantifications.

### Quantitative real-time PCR analysis

Total RNA was isolated from harvested cells using the RNA Blood Kit (Qiagen) following the manufacturer’s instructions^40^. Reverse transcription was performed using the SuperScript VILO cDNA Synthesis Kit (Invitrogen, Thermo Fisher Scientific) and the cDNA was purified using the PCR Purification Kit (Qiagen). TaqMan^®^ Gene Expression Assays (Applied Biosystems, Thermo Fisher Scientific) were used to perform quantitative real-time PCR of *BIP* (Hs99999174_m1), *CHOP* (Hs99999172_m1), and *SLC35B1* (Hs00195184_m1) in a StepOne Plus 96-well system (Applied Biosystems). The Δct-values were calculated using *ACTB* (Hs00357333_m1) as a standard, and the values were normalized based on control siRNA-treated cells.

### Live-cell ATP imaging

Live-cell imaging of ATP in the cytosol and ER lumen was performed using the genetically encoded ATP sensors ATeam^33,41^ and ERAT4.01^25^, respectively. *SLC35B1* silencing and expression plasmid based complementation in HeLa cells was performed for 96 h as described above. In addition, 24 h before measurement (72 h after the first siRNA transfection), the cells were transfected with 1 µg of the plasmids encoding the respective ATP sensor using Fugene HD. Then, 8 h after the last transfection, cells were seeded on 25 mm glass coverslips. Prior to the ATP measurements, the cells were incubated at room temperature for at least 2 h in loading buffer^25^ (135 mM NaCl, 5 mM KCl, 2 mM CaCl_2_, 1 mM MgCl_2_, 20 mM HEPES, 2.6 mM NaHCO_3_, 0.44 mM KH_2_PO_4_, 0.34 mM Na_2_HPO_4_, 10 mM D-glucose). Coverslips were placed in a perfusion chamber, and imaging was performed at room temperature in Ringer’s buffer (145 mM NaCl, 4 mM KCl, 2 mM MgCl_2_, 2 mM CaCl_2_, 10 mM Hepes, 10 mM glucose, pH 7.4). Cells were imaged using a Cell Observer High Speed microscope (Zeiss) with a ×40 oil Fluar objective (Zeiss) and an Evolve 512 EMCCD camera (Photometrics). The sensors were excited using 420 nm, 505 nm, and white light LEDs (Colibri, Zeiss), while emission light was collected with CFP (Semrock HC) and YFP (Zeiss) single-band filters. Images of all channels were acquired every 3 s, and FRET-ratios were calculated with AxioVision Software (Zeiss). Background fluorescence was subtracted, and the values were corrected for bleed-through and bleaching. Data were analyzed using Microsoft Excel 2013. *P* values were determined using unpaired *t*-tests.

### Live-cell Ca^2+^ imaging

HeLa cells that were treated as described above were loaded with 4 µM Fura-2 AM in DMEM and incubated for 30 min at room temperature^6,27^. The cells were washed twice and incubated at room temperature in Ca^2+^-free buffer (140 mM NaCl, 5 mM KCl, 1 mM MgCl_2_, 0.5 mM EGTA, 10 mM glucose in 10 mM HEPES-KOH, pH 7.35). Ratiometric measurements were performed for 5 or 10 min using an iMIC microscope and the polychromator V (Till Photonics) with alternating excitation at 340 nm and 380 nm and measurement of the fluorescence emitted at 510 nm. The microscope was equipped with a Fluar M27 lens with ×20 magnification and 0.75 numerical aperture (Carl Zeiss) and an iXon^EM^ + camera (Andor Technology). Images containing 50–55 cells/frame were sampled every 3 s using TILLvisION software (Till Photonics). Fura-2 signals were recorded as the F340/F380 ratio, where F340 and F380 correspond to the background-subtracted fluorescence intensities at 340 nm and 380 nm, respectively. Data were analyzed using Microsoft Excel 2007. *P* values were analyzed using unpaired *t*-tests.

### Reconstitution of membrane proteins and ATP transport

Rough microsomes were isolated from canine or porcine pancreas and converted to ribosome-depleted microsomes (PKRM) by treatment with puromycin and high salt^16^. Alternatively, bacterial membranes from SLC35B1 expressing cells were isolated as described above. Proteoliposomes were prepared using 100 mg/ml phosphatidyl choline (type IV-S from soya bean; Sigma) and sonicated in 30 mM potassium gluconate, 100 mM Tricine-NaOH (pH 7.5)^42^. Liposomes were subsequently preloaded with 10 mM counter-exchange nucleotides (except when otherwise stated). PKRM and bacterial proteins were solubilized with 1% Triton X-100 and 1% dodecylmatoside, resepectively, for 1 min on ice. The samples were centrifuged (1 min, 15,800×*g* at 4 °C) and the supernatants were rapidly combined with the liposomes and mixed vigorously (resulting detergent concentration 0.1%). The vigorously mixed suspension was subsequently transferred to liquid nitrogen to allow incorporation of the proteins into the vesicles after thawing the sample at 4 °C (freeze-thaw method). The thawed proteoliposomes were sonicated for 30 s (20% line voltage, 20% duty cycle) on ice. Unincorporated solution was removed by passing the proteoliposomes over NAP-5 gel filtration columns pre-equilibrated with 150 mM potassium gluconate, 10 mM Tricine-NaOH, pH 7.5 at 4 °C. The eluted proteoliposomes (1 ml) were immediately used for transport measurements. Transport at 30 °C was started by adding 100 µl proteoliposomes to 100 µl transport medium (150 mM potassium gluconate, 10 mM Tricine-NaOH, pH 7.5) containing the indicated [^32^P]-labeled substrates. Uptake occurred at the indicated time spans and was stopped by transferring the liposomes to a 1 ml Dowex AG-1 (Cl^−^ form; 100–200 mesh) column pre-equilibrated with 200 mM Tricine-NaOH pH 7.5. By washing the columns with 3 × 500 µl ice cold equilibration medium the proteoliposomes were eluted and external radioactivity could be removed. Imported radioactivity was quantified in a scintillation counter.

### AXER modeling and ATP docking

The comparative atomic model of SLC35B1 was modeled using two different computational approaches: Phyre2^34^ and RaptorX^43^. The structure predicted by Phyre2 is very similar to that of the DMT superfamily transporter YddG (PDB ID: 5I20) with an RMSD of 0.4 Å. On the other hand, the structure of SLC35B1 predicted by RaptorX was based on that of the triose-phosphate/phosphate translocator (PDB ID: 5Y78)^44^. It has a RMSD of 4.8 Å of RMSD compared to the template. The two models show a similar topological arrangement of the transmembrane helices. The RMSD difference between the two models is 6.97 Å. A residue contact map was derived by the RaptorX Contact Prediction method^45^ to validate the two models obtained in the previous step. The validation showed that 59% of the predicted contact pairs are fulfilled by the Phyre2 model (only considering contacts with more than 90% probability in the contact map). For comparison, 81% of the predicted contact pairs are fulfilled by the RaptorX model. However, as the RaptorX model adopts a closed structure, we considered the open Phyre2 to be more promising for a ligand docking study.

Hence, the model obtained by Phyre2 was subjected to energy minimization by the NAMD package to relax the side-chain atoms. Subsequently, the minimized structure was subjected to the ProSa-web server for structure validation. The ProSa *Z*-score of −3.03 is in the typical range or scores found for experimentally determined structures of protein chains. The ATP molecular structure was retrieved from the PubChem database. Protonation states at neutral pH were assigned with the Babel software. The resulting structure of ATP had the expected total charge of −4e. After setting up protein and ligand, docking runs were carried out using the AutoDock4 program (2.5 × 10^6^ energy evaluations and 27 × 10^3^ generations) to scan for energetically favorable conformations of the ligand inside the protein pocket.

### Data availability

Source files for Western blots were deposited at Mendeley (10.17632/b49tkfjcm5.1). In addtion, all data are available from the authors.

## Electronic supplementary material

Supplementary Information

Peer review_File
