# Supplementary material for: AXER is an ATP/ADP exchanger in the membrane of the endoplasmic reticulum
Source: Nat Commun. 2018 Aug 28;9:3489. doi: 10.1038/s41467-018-06003-9 (PMC6113206; doi:10.1038/s41467-018-06003-9)
Supplement: Supplementary file 1 — Supplementary Information [file 41467_2018_6003_MOESM1_ESM.pdf]

Supplementary Information for

AXER is an ATP/ADP exchanger in the membrane of the endoplasmic reticulum

Klein *et al.*

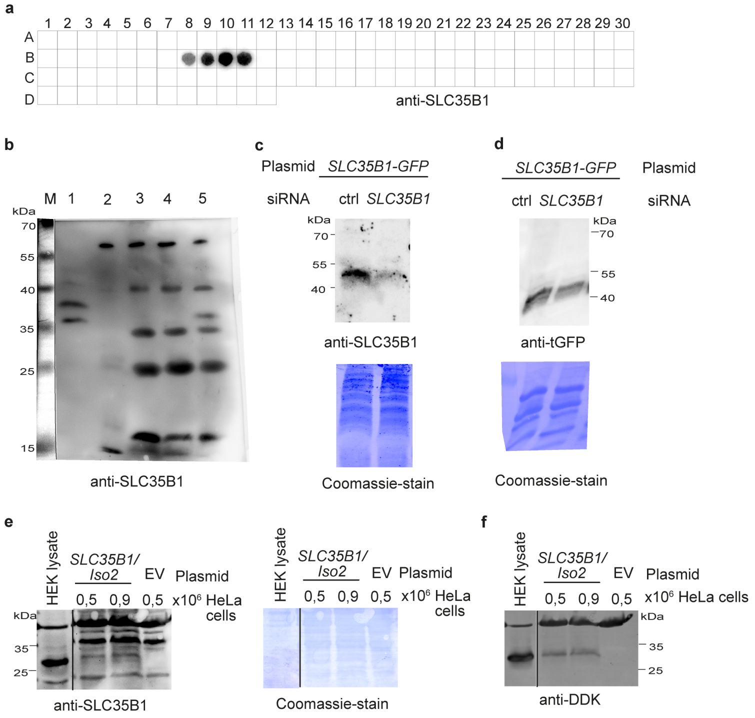

Supplementary Figure 1 | Detection of endogeneous and over-produced SLC35B1 in canine pancreas and HeLa cells. (**a**) SLC35B1 peptide array for the characterization of anti-SLC35B1 antibody. 102 peptides comprising 20 consecutive amino acids of SLC35B1 and over-lapping by 17 amino acids with the next peptide were synthesized as distinct spots onto cellulose membranes as described previously[6,27]. The membranes were blocked[27] and probed with SLC35B1-specific antibody. SLC35B1-specific antibody was visualized using POD-conjugated secondary antibodies and ECL™. The chemiluminescence image of the blot is shown. **(b)** Detection of SLC35B1 in extracts from mammalian rough microsomes. A 4% digitonin extract of canine pancreatic rough microsomal membrane proteins (derived from 6 mg microsomal protein) was subjected to SDS-PAGE in parallel to *E. coli* membranes (25 µg protein), which were derived from non-transfected and SLC35B1- or SLC35B1/isoform 2-expressing cells. The Western blot was decorated with SLC35B1-specific antibody and visualized with peroxidase-coupled secondary antibodies, Super Signal West Pico, and luminescence imaging. A molecular mass standard (M) was run in parallel and electronically copied from the stained blot to the Western blot image. The area of interest of this blot is

shown in Fig. 1c. (**c-f**) Detection of over-produced SLC35B1 in HeLa cells. HeLa cells were transfected with empty vector (EV) or *SLC35B1* expression plasmids, encoding either SLC35B1-GFP or My-DDK-tagged SLC35B1/Isoform 2, for 48 h. Cells were analysed by SDS-PAGE and Western blotting using either anti-SLC35B1-, anti-GFP-, or anti-Myc-DDK-antibodies. The first two antibodies were visualized with peroxidase-coupled secondary antibodies, Super Signal West Pico, and luminescence imaging, the third one with ECL™ Plex goat anti-mouse IgG-Cy3 conjugate using the Typhoon-Trio imaging system. Coomassie staining of the blots for total protein served as a loading controls. (**c, d**) 0.5 x 10^6 SLC35B1-GFP producing HeLa cells were subjected to SDS-PAGE. The Western blots were decorated with either anti-SLC35B1 (**c**) or anti-tGFP (**d**). (**e, f**) The indicated number of Myc-DDK-tagged SLC35B1/Isoform 2 producing HeLa cells (0.5 – 0.9 x 10^6, corresponding to 300-540 µg protein) were subjected to SDS-PAGE in parallel to a commercially available cell lysate from strongly Myc-DDK-tagged SLC35B1/Isoform 2 producing HEK293 cells (13.3 µg protein, positive control) and non-transfected HeLa cells (0.5 x 10^6, corresponding to 300 µg protein, negative control). The Western blots were decorated with either anti-SLC35B1 (**e**) or anti-Myc-DDK (**f**).

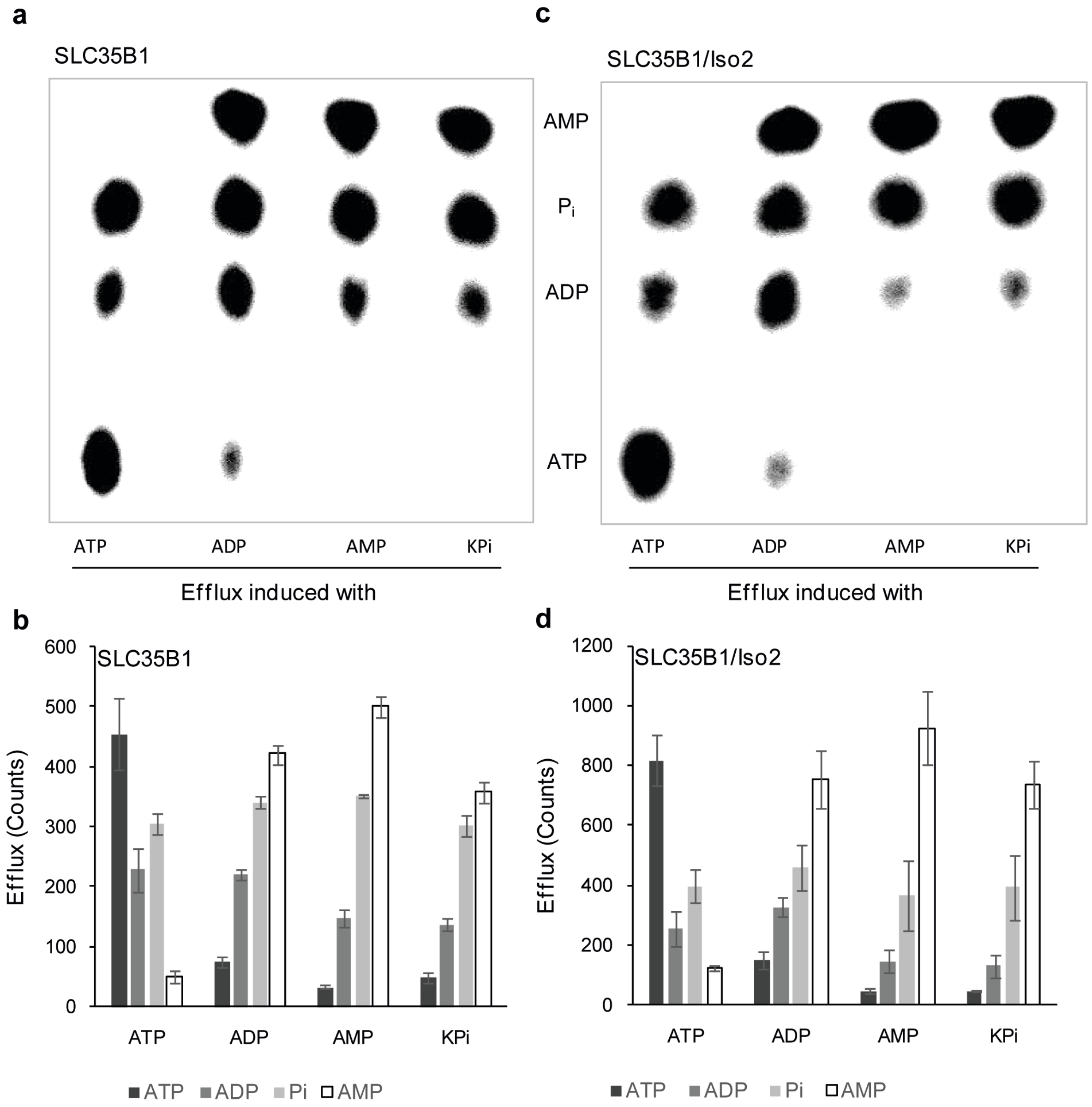

Supplementary Figure 2 | Exchange-mediated efflux of intracellular ATP by SLC35B1. Due to metabolic activity of *E. coli* cells [^{32}P]ATP is converted into labeled ADP, AMP, and inorganic phosphate (P_i), the chase of labeled ATP from the transfected cells was incomplete (Fig. 3a, b). (**a-d**) To nevertheless characterize this chase reaction as such, SLC35B1 (**a, b**) and SLC35B1/Isoform 2 (**c, d**) expressing *E. coli* cells were loaded with 50 µM [^{32}P]ATP for 5 min[9]. Non-imported radioactivity was removed by washing (three times in phosphate buffer). Subsequently, cells were incubated in phosphate buffer with 500 µM ATP, ADP or AMP or without adenine nucleotides (KP_i) as indicated. Efflux was allowed for 3 min and terminated by rapid centrifugation. Ten microliters of the supernatant were chromatographed

on a 0.5-mm poly(ethylene amine) cellulose thin-layer plate, dried with a fan, and subjected to autoradiography. The nature of exported label was identified by comparison with radioactively-labeled standards (indicated in the center). Radioactively-labeled positions were marked on the thin-layer plate, cut out, and quantified in a scintillation counter. A representative autoradiogram is shown in **a** and **c**. Values in **b** and **d** are the mean of three independent experiments. The results support the conclusion that SLC35B1 and SLC35B1/Isoform 2 act as antiporters.

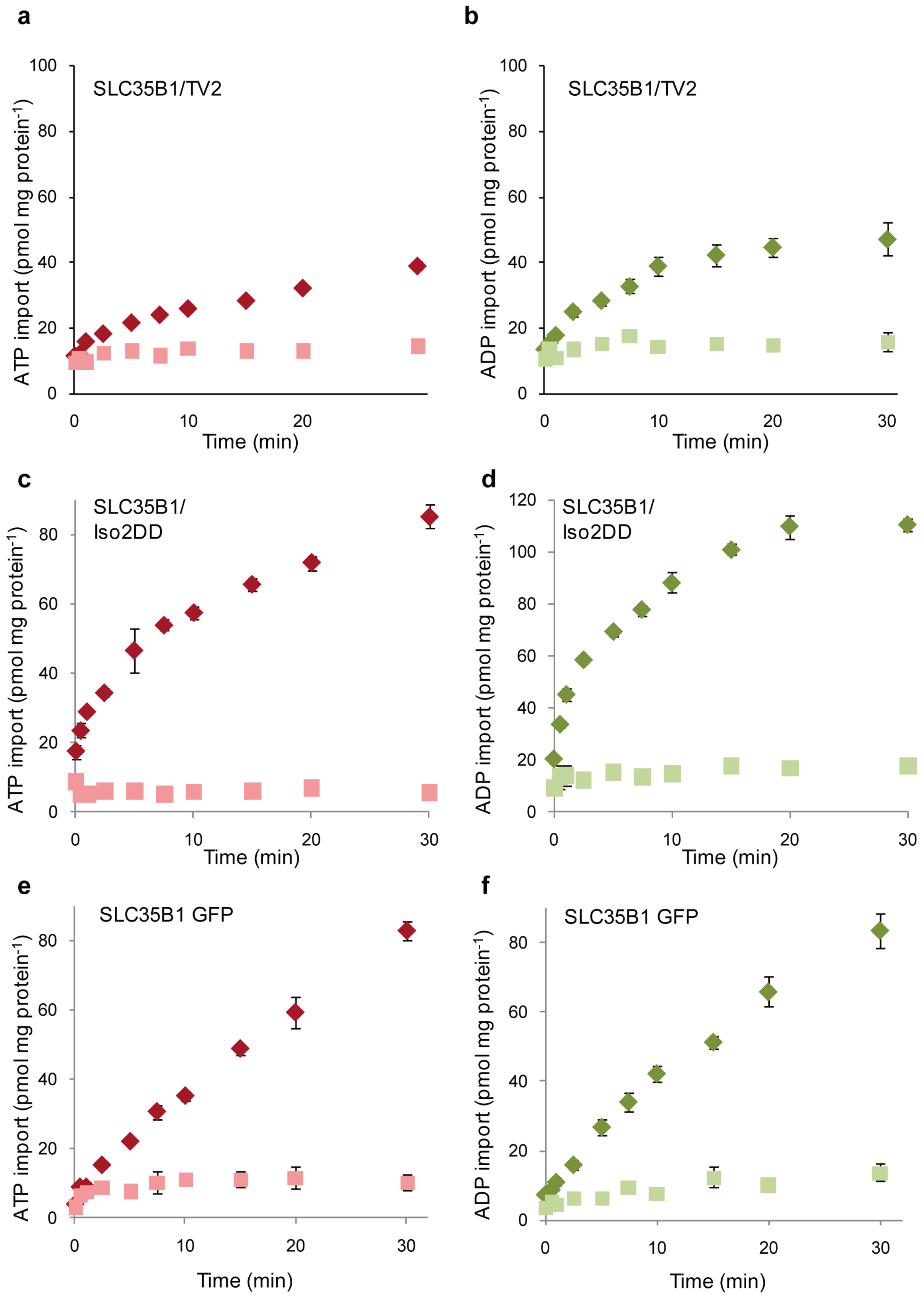

Supplementary Figure 3 | Heterologously expressed variants of SLC35B1 are ATP and ADP carriers. (**a-f**) The indicated SLC35B1 variants, which were described and shown in Fig. 2a, were expressed in different *E. coli* cells. Uptake of 50 µM [α^{32}P]ATP (**a, c, e**) or

[α^{32}P]ADP (**b, d, f**) into *E. coli* cells which were expressing either SLC35B1 transcript variant 2 (TV2) (**a, b**), the phosphomimetic variant of SLC35B1/Isoform 2 with aspartate residues at positions 15 and 29 instead of serines, termed SLC35B1/Isoform 2DD (**c, d**), or SLC35B1-GFP (**e, f**), was measured and compared to uptake by non-induced cells.

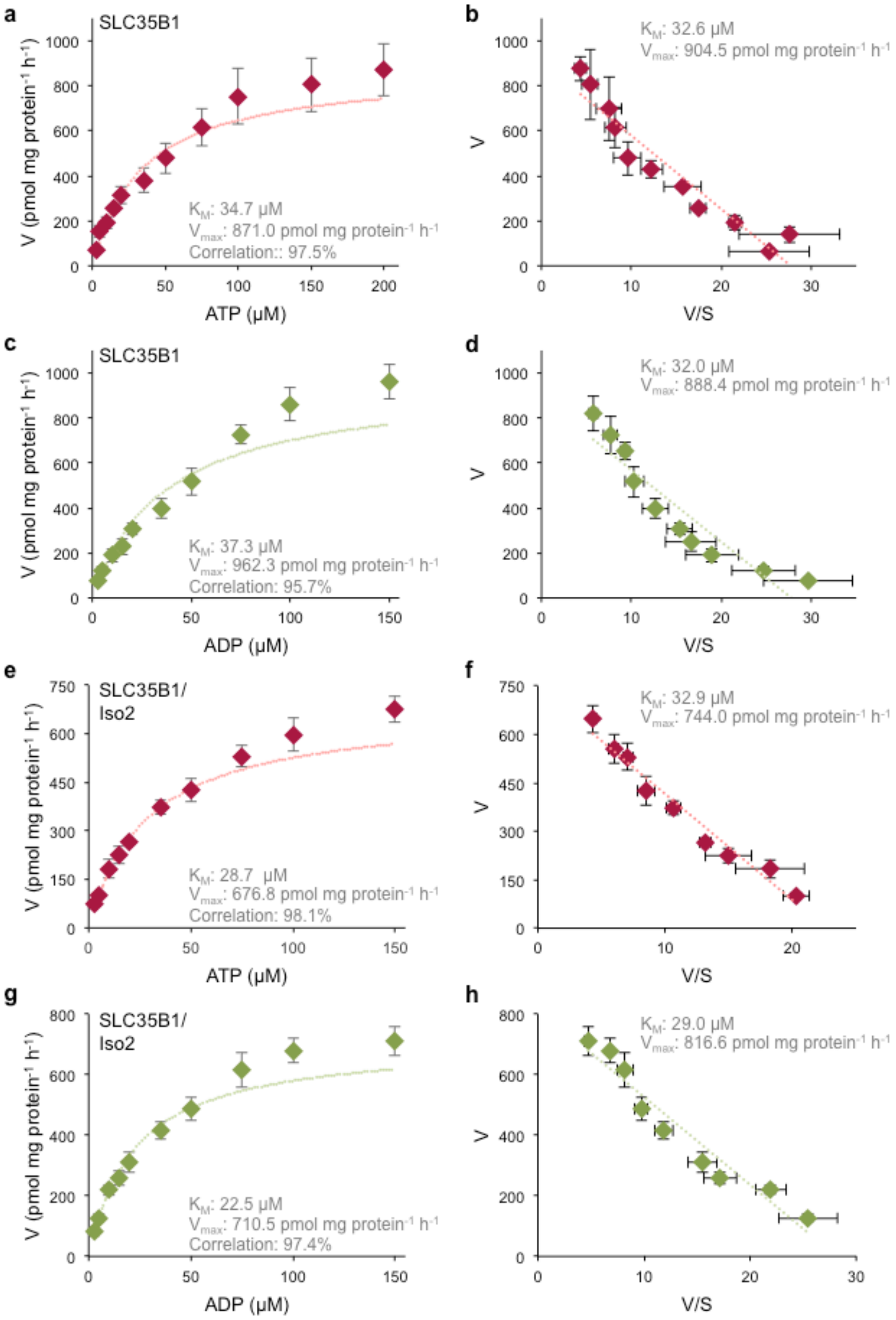

Supplementary Figure 4 | Determination of K_M and V_{max} values for ATP and ADP of heterologously expressed SLC35B1. To reveal the apparent affinity of the heterologously

expressed SLC35B1 for ATP or ADP substrate saturation experiments were performed. Rising concentrations of exogenous [α^{32}P]-ATP and ADP led to an increase of import activity approaching apparent saturation above 120 µM. Non-linear curve fitting and Eadie-Hofstee analyses of this saturation curve revealed the apparent K_M values and maximal uptake rates (V_{max}), which are indicated. For non-linear curve fitting the data were fitted to the Hill equation $y = V_{max} \times x^n/(k^n + x^n)$ with $n = 1$ for noncooperative binding. Calculations were performed with Origin 8 (OriginLab Corp.). **(a-h)** SLC35B1 (**a-d**) or SLC35B1/Isoform 2 (**e-h**) were expressed in *E. coli* and uptake of [α^{32}P]ATP (**a**, **b, e, f**) or [α^{32}P]ADP (**c, d, g, h**) was measured. Data are reported as the mean of at least three independent experiments and are shown with the standard error of the mean (SEM).

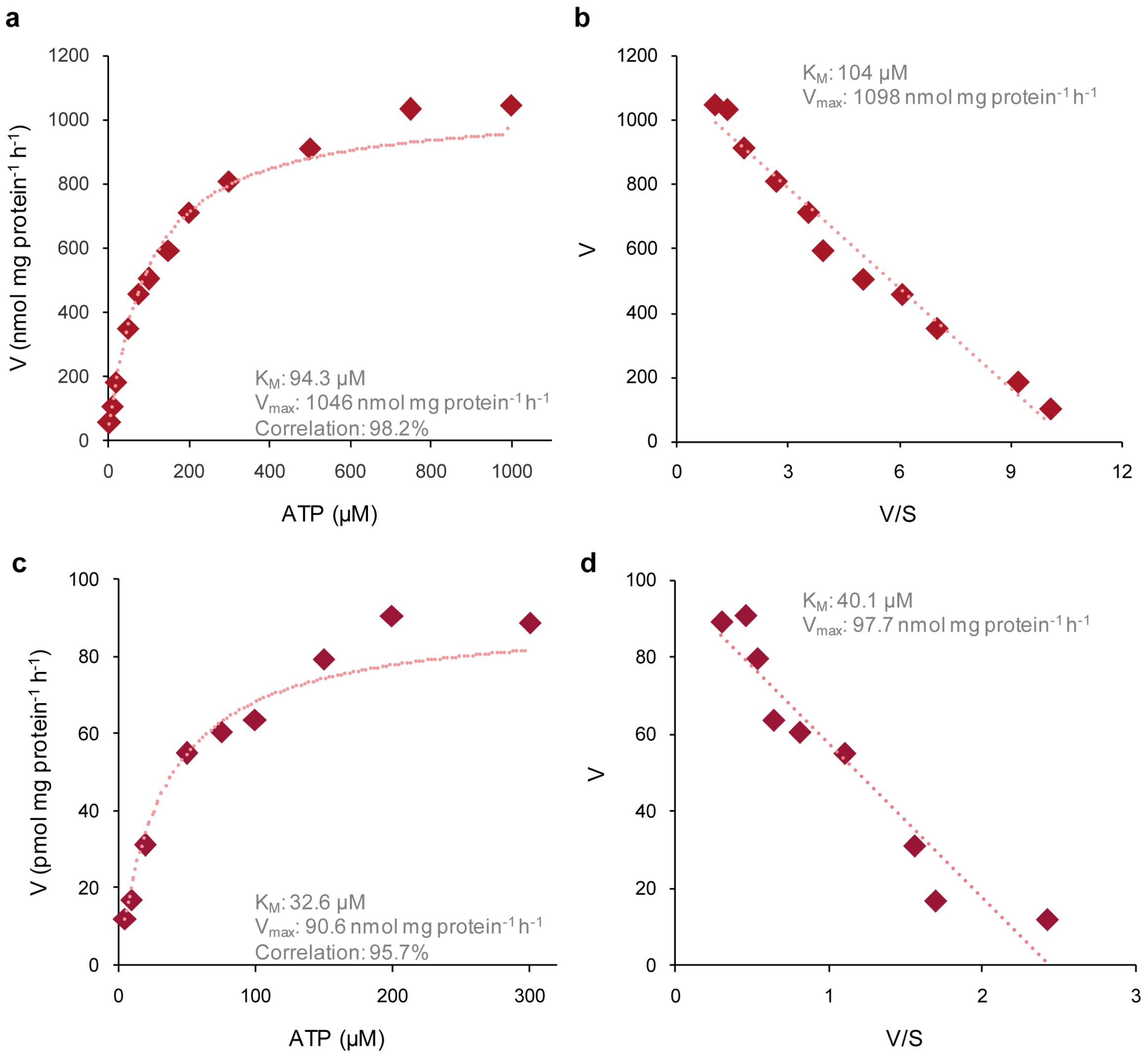

Supplementary Figure 5 | Determination of K_M and V_{max} values for ATP and ADP of ATP transport into proteoliposomes with the full complement of mammalian ER. Proteoliposomes harbouring porcine or canine pancreatic rough ER membrane proteins were prepared as described in Methods. To reveal the apparent affinity of the ER nucleotide transporter for ATP and ADP substrate saturation experiments were performed. Substrate saturation of [α^{32}P]-ATP in preloaded (10 mM ADP) proteoliposomes, which were derived from porcine (**a**) or canine (**c**) pancreatic microsomes. Uptake was terminated after 2 min. Rising concentrations of exogenous [α^{32}P]-ATP led to an increase of import activity approaching apparent saturation above 500 µM. For ATP, non-linear curve fitting and Eadie-Hofstee analyses (**b**, **d**) of these saturation curves revealed the apparent K_M values and

maximal uptake rates (V_{max}), which are indicated. For non-linear curve fitting the data were fitted to the Hill equation $y = V_{max} \times x^n/(k^n + x^n)$ with $n = 1$ for noncooperative binding. Calculations were performed with Origin 8 (OriginLab Corp.). Similar results were obtained for ADP: proteoliposomes from porcine microsomes (preloaded with ATP) exhibited K_M and V_{max} values of 80.7 μM and 560.8 nmol mg protein^{-1} h^{-1}; proteoliposomes from canine microsomes (preloaded with ATP) exhibited K_M and V_{max} values of 33.6 μM and 44.1 nmol mg protein^{-1} h^{-1}. The data are reported as the mean of at least three independent experiments. We note that the proteoliposomes harbouring canine pancreatic rough ER membrane proteins were prepared from a similar detergent extract, as described in the legend to Fig. 1c.

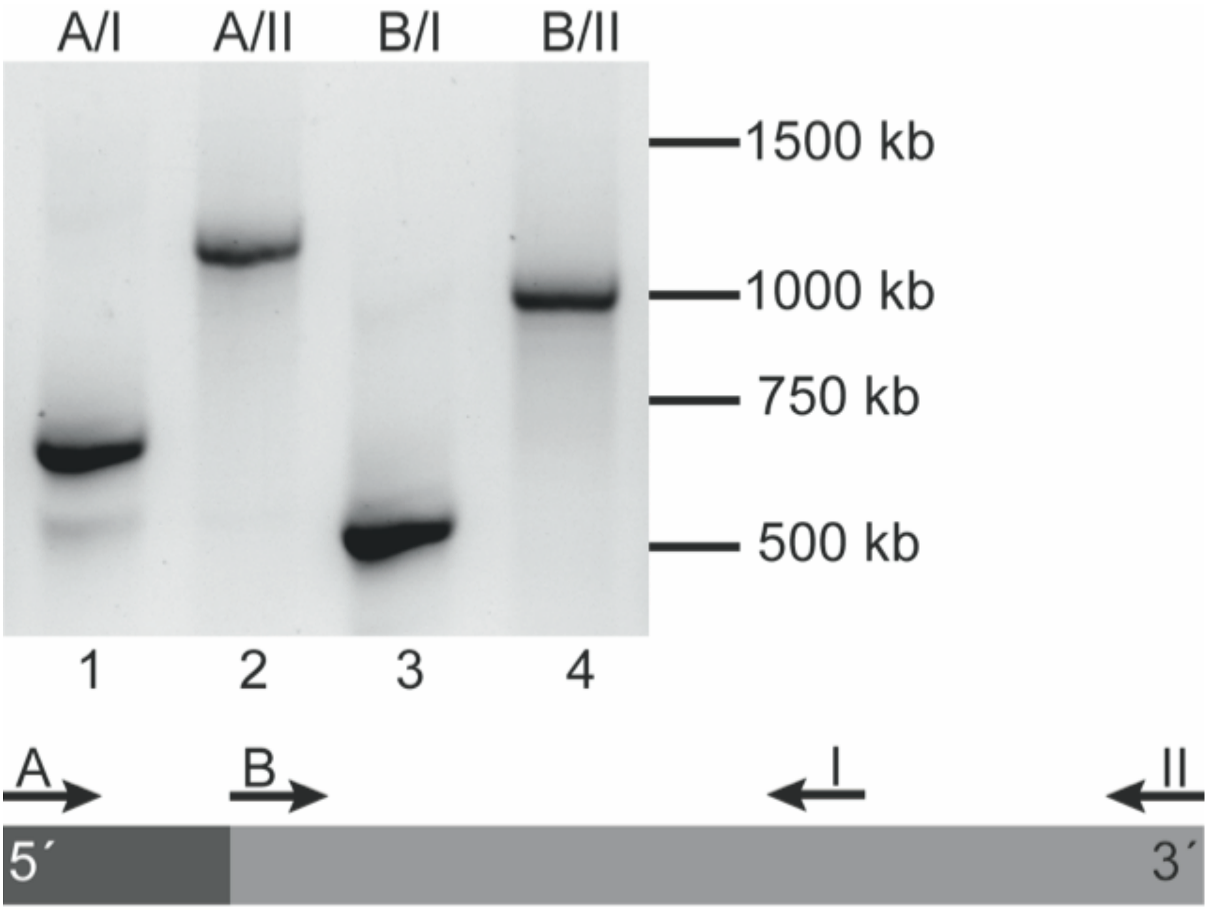

Supplementary Figure 6 | Detection of *SLC35B1* mRNA in HeLa cells. The same cDNA, which was used for qRT-PCR experiments and is described in Methods, was used to adress the question which *SLC35B1* mRNA variants are present in HeLa cells. Briefly, the cDNA was subjected to amplification using the Phusion High-Fidelity Polymerase (Thermo Fisher Scientific) with four different primer combinations according to the manufacturer´s recommendations in a primus advanced 96 thermocycler (PEQLAB). The primers were purchased from eurofins (A, 5´ATGAGGCCCCTGCCGCCGGTC3´, B, 5´ATGGCCTCTAGCAGCTCCCTG3´, I, GGGTCAGCGATAATAGCAAGA3´, II, 5´ACGCGTGTGGGATGTCTTCTTAG3´). (**a**) The PCR products were subjected to agarose gel electrophoresis and stained with GelRed Nucleic Acid Stain (Biotium). The relevant part of the gel is shown with size markers. (**b**) The positions of the primers on the cDNA are shown. The dark area highlights the coding region of the 37 amino acid residues that differentiate between Isoforms 1 and 2.

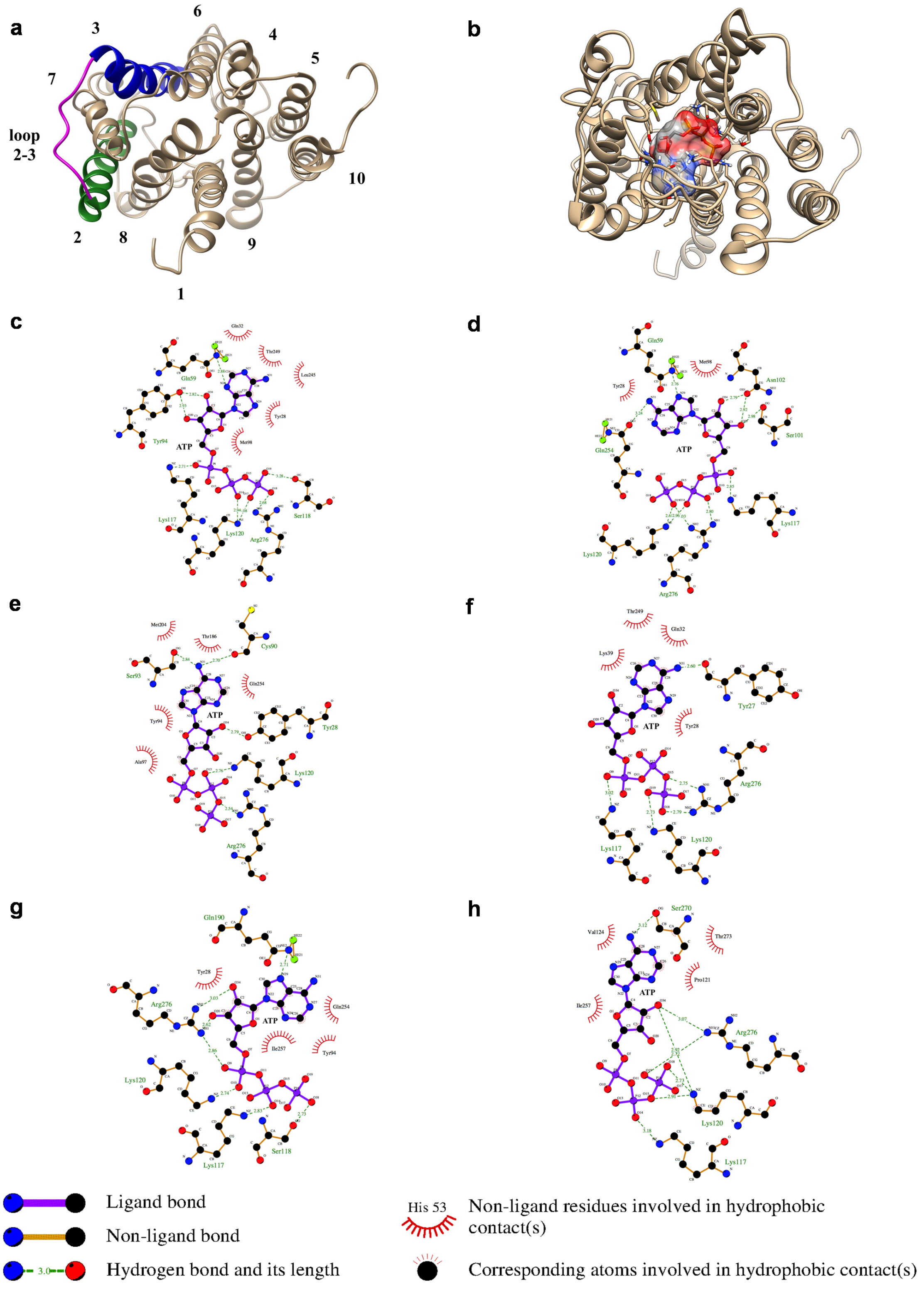

Supplementary Figure 7 | Binding sites of ATP in SLC35B1. (a, b) A hypothetical structural model of the human SLC35B1, as predicted by the Phyre2 server[34] and seen from

the cytosol (**a**) or the ER lumen (**b**). (**a**) Helices 2 (green) plus 3 (blue) and the connecting loop (purple) with the putative IQ motif are highlighted as in Fig. 1b. (**b**) ATP was docked into the predicted structure using the AutoDock4 program to scan for energetically favorable conformations of the ligand inside the protein pocket. Subsequent clustering of the obtained results based on RMSD suggested six favorable binding modes of ATP inside SLC35B1. Shown is the best-ranked conformation of ATP with a predicted binding free energy of -11.33 kcal/mol. (**c-h**) Predicted contacts between AT and amino acid residues of the six top-ranked docking poses are illustrated with the LigPlot software. In these poses, the negative phosphate groups are coordinated by the same set of positively charged amino acid residues (Lys 117, Lys 120, Arg 276), the H-bonding atoms of the adenine ring are coordinated by polar groups, and the adenine ring is positioned close to aromatic residues (Tyr 28, Tyr 94). Such contacts are typical coordination scenarios of ATP bound to proteins. Hence, all six docking poses can be considered as plausible binding modes.

a

MRPLPPVGDVRLELSPPPPLLPVPVVSGSPVGSSGRL
MASSSSLVPDRLRLPLCFLGVFVCYFYYGILQEKITRGKYGEGAKQETFT
FALTLVFIQCVINAVFAKILIQFFDTARVDRTRSWLYAACSISYLGAMVS
SNSALQFVNYPTQVLGKSCKPIPVMLLGVTLLKKKYPLAKYLCVLLIVAG
VALFMYKPKKVVGIEEHTVGYGELLLLLSLTLDGLTGVSQDHMRAHYQTG
SNHMMLNINLWSTLLLGMGILFTGELWEFLSFAERYPAIIYNILLFGLTS
ALGQSFIFMTVVYFGPLTCSIITTTRKFFTILASVILFANPISPMQWVGT
VLVFLGLGLDAKFGKGAKKTSH

b

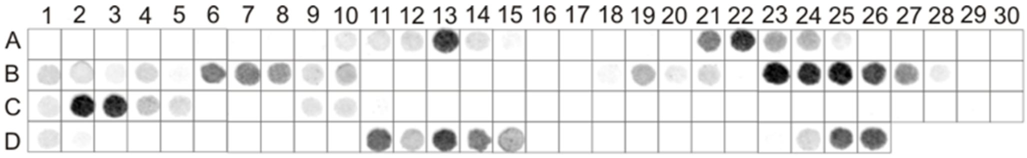

Supplementary Figure 8 | Binding sites of Ca^{2+}-CaM in SLC35B1. (a) Amino acid sequence of SLC35B1 Isoform 2 with binding site for Ca^{2+}-CaM, as deduced from peptide

array shown in **b**. The indicated physical binding site of Ca^{2+}-CaM (shown in green) coincided with the computationally predicted Ca^{2+}-CaM binding site (shown in yellow) (http://calcium.uhnres.utoronto.ca/ctdb/ctdb/sequence.html) and is located in the cytosolic loop connecting transmembrane helices 2 and 3 (Fig. 1b, Supplementary Fig. 7a). The additional binding sites coincided or at least overlapped with transmembrane domains, probably due to the known affinity of the ligand with hydrophobic peptides. (**b**) 116 peptides comprising 15 consecutive amino acids of SLC35B1 Isoform 2 and overlapping by 12 amino acids with the next peptide towards the carboxy terminus were synthesized as distinct spots onto cellulose membranes as described previously[6,27]. The membranes were blocked and probed with ^{14}C-labelled Ca^{2+}-CaM[27] as described before. ^{14}C-labelled Ca^{2+}-CaM was detected by Phosphorimaging. We note that binding of ^{14}C-labelled Ca^{2+}-CaM to all spots was prevented by the presence of EGTA and to the binding site in spots B6-8 (shown in green in **a**) by mutation of either one of arginine residues 4, 7 or 9 to alanine.

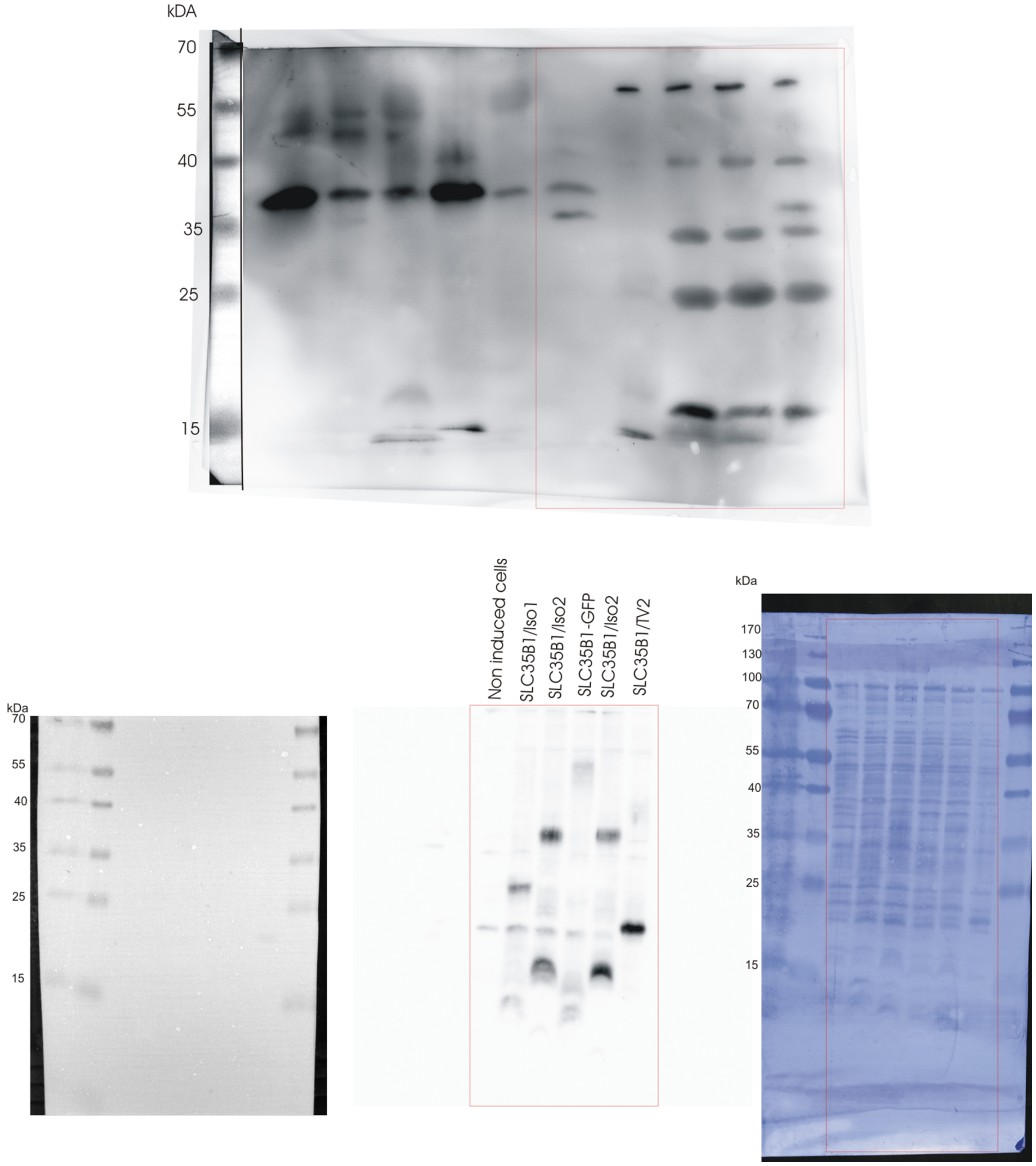

Supplementary Figure 9 | Full scans for Western blots and stained blots, respectively, which are shown in Fig. 1c, Supplementary Fig. 1b, and Fig. 2a and b.

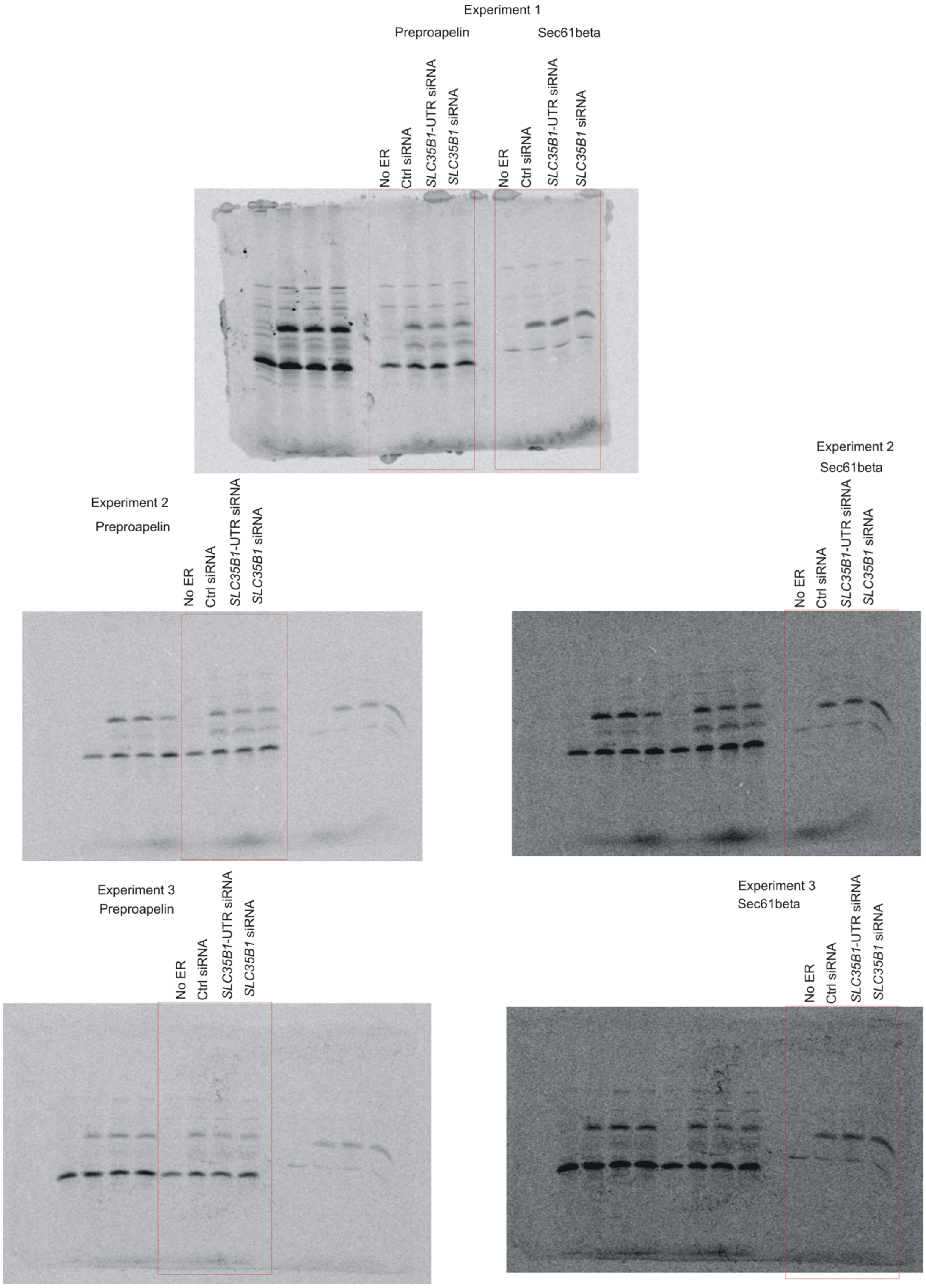

Supplementary Figure 10 | Full scans for phosphorimager images, which are shown in Fig. 8a-d.

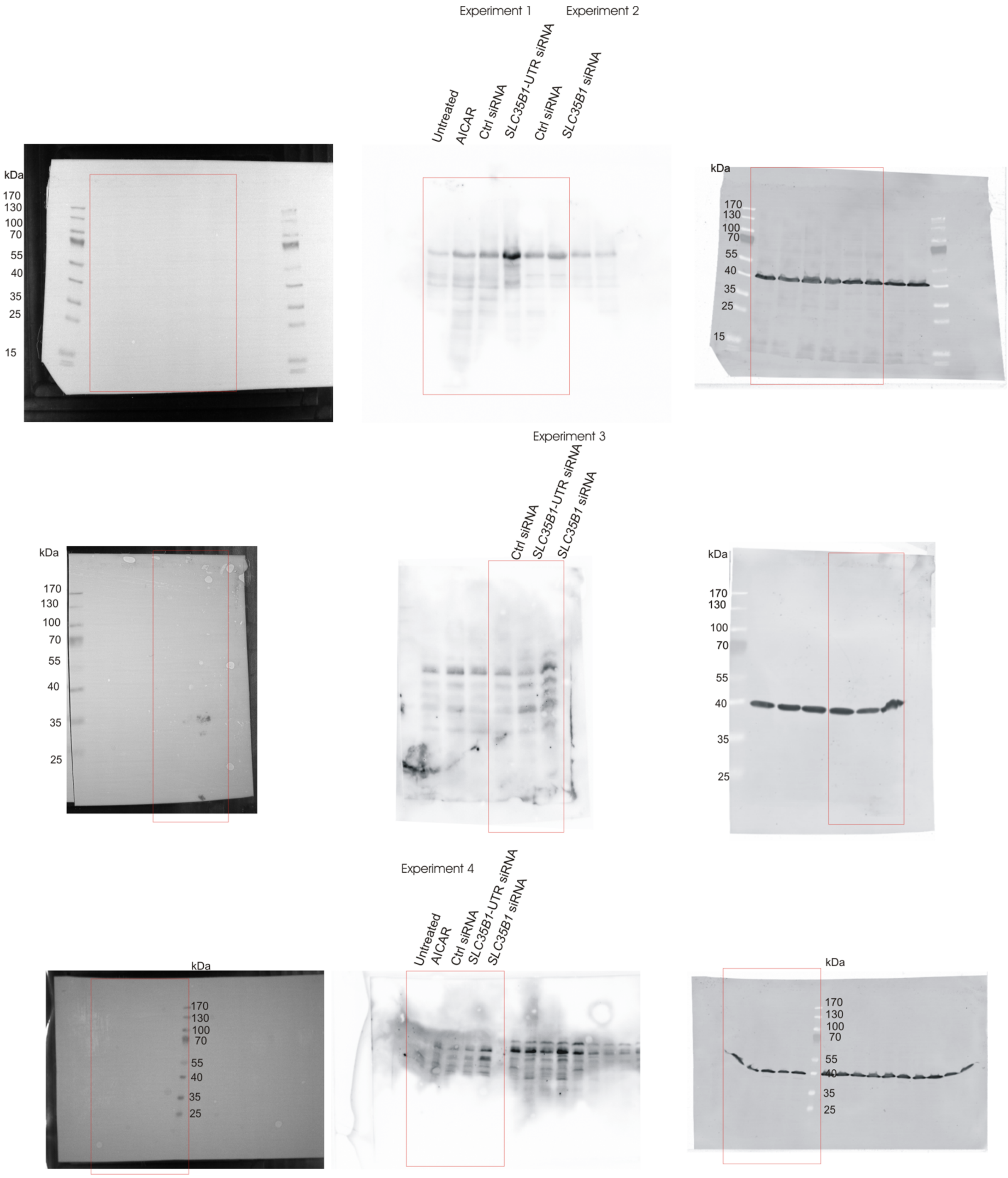

Supplementary Figure 11 | Full scans for Western blots and stained blots, respectively, which are shown in Fig. 8m-n.

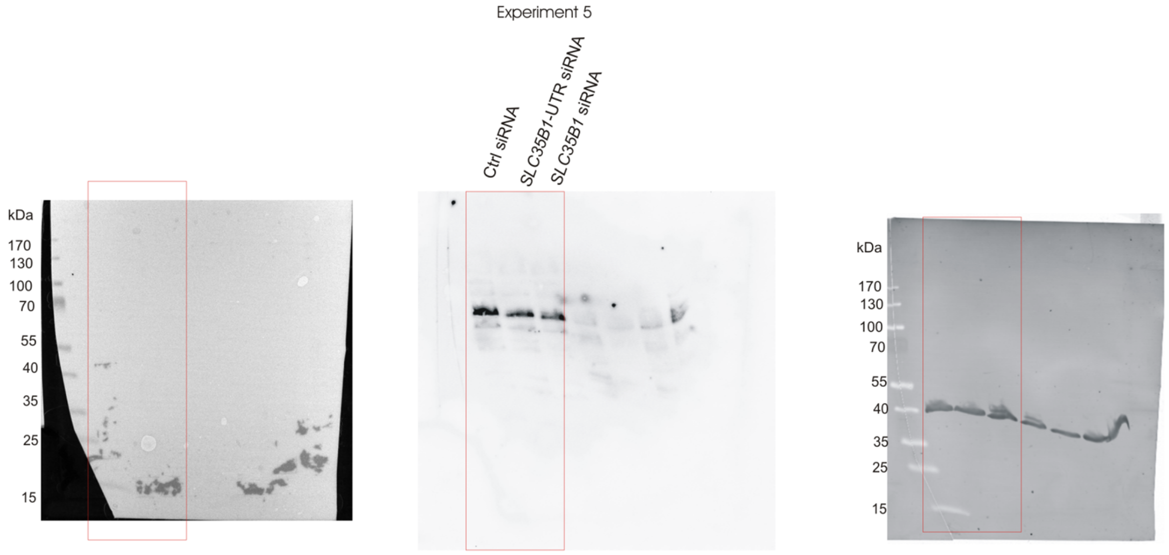

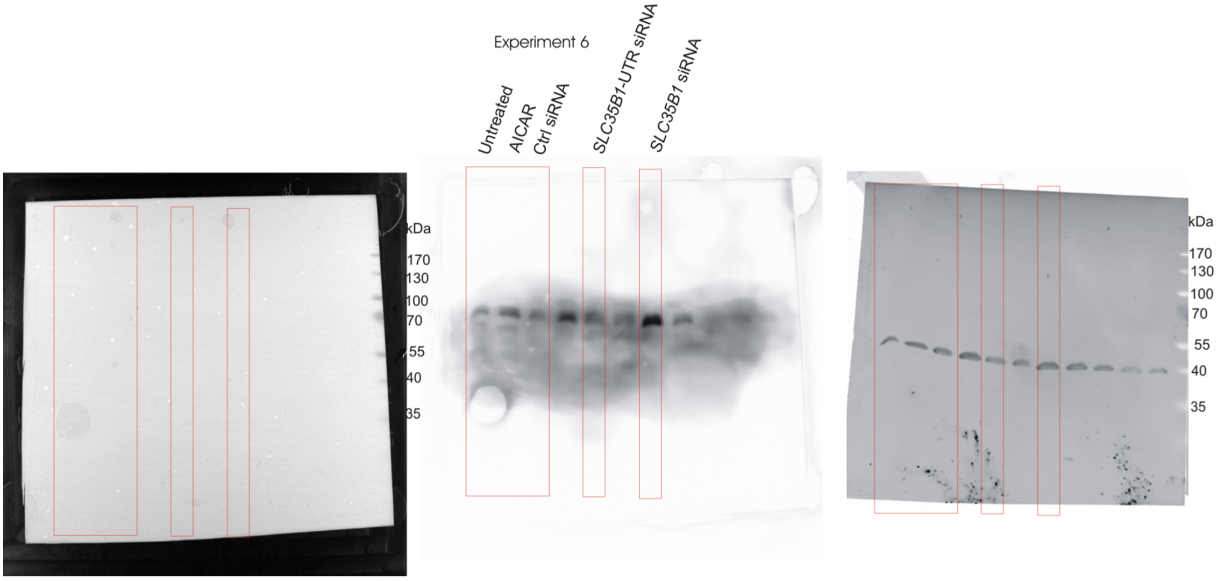

Supplementary Figure 12 | Full scans for Western blots and stained blots, respectively, which are shown in Fig. 8m-n.

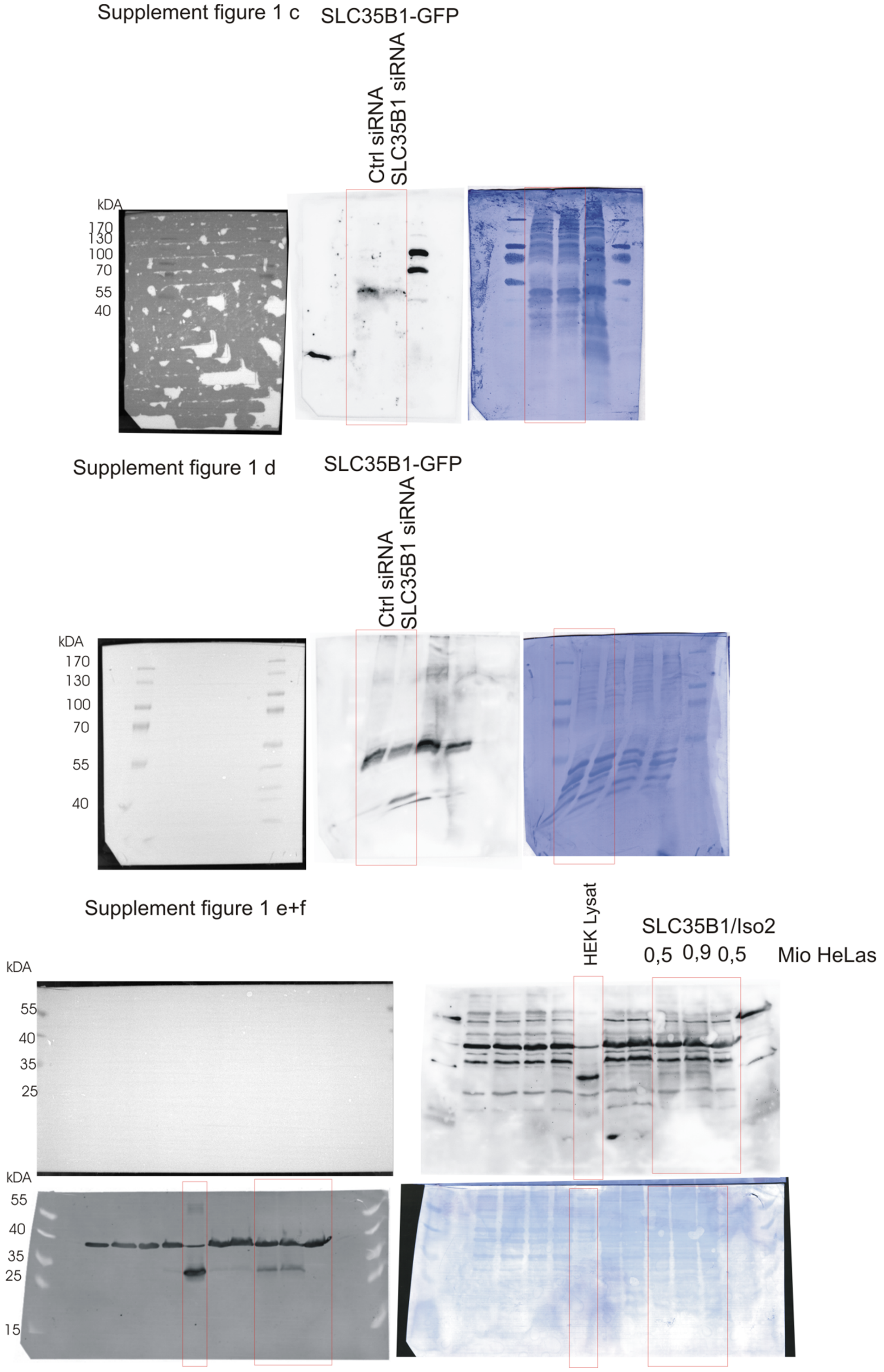

Supplementary Figure 13 | Full scans for Western blots and stained blots, respectively, which are shown in Supplementary Fig. 1c-f.

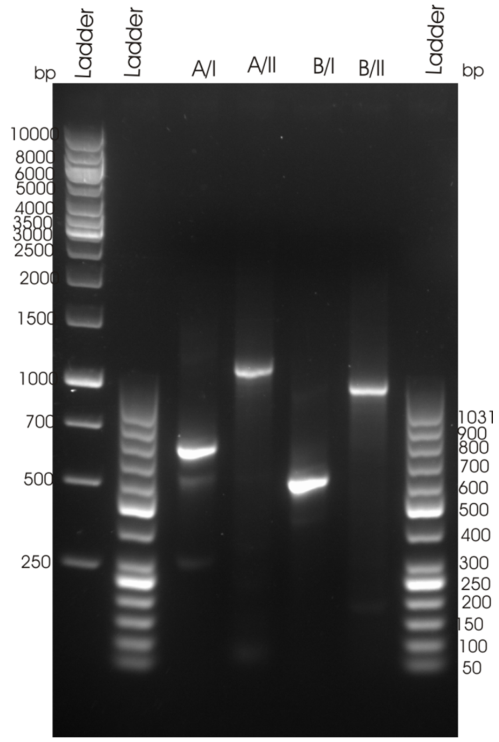

Supplementary Figure 14 | Full scans for imaged agarose gel which is shown in Supplementary Fig. 6.

Supplementary Table 1 | Protein content in HeLa cells.

Protein	Gene	Copy Number[17]	LFQ intensity
AMPK alpha 1	*PRKAA1*	147,133	1,272,900,000
AMPK beta 1	*PRKAB1*	277,161	239,796,000
AMPK gamma 1	*PRKAG1*	96,958	613,954,000
BiP (Grp78)	*HSPA5*	9,940,200	112,141,666,000
Calmodulin 1 (CaM)	*CALM1*	11,354,804	15,061,333,000
CAMKK2	*CAMKK2*	18,969	75,197,000
6-Phospofructo-2-kinase	*PFKFB2*	189,153	512,943,000
PP2AA	*PPP2CA*	1,413,926	433,383,000
PP2AB	*PPP2CB*	89,902	162,930,000
Sec61 alpha	*SEC61A1*	167,488	1,305,500,000
Sec61 beta	*SEC61B*	549,535	1,903,083,000
Sec61 gamma	*SEC61G*	482,107	852,783,000
SLC35B1 (UGTREL1)	*SLC35B1*	21,210	15,832,000

Copy numbers were obtained from the literature[17]. Label-free quantification (LFQ) intensities were obtained by whole proteome analysis of HeLa cells. Notably, LFQ intensities do not reflect true copy numbers because they depend not only on the amounts of the peptides but also on their ionization efficiencies[17]; thus, they only serve as an indication of the presence of the protein of interest.

Supplementary Table 2 | SLC35B1 interactome in HeLa cells.

#	Identified protein	Accession #	MW (kDa)	Sum
1	ATP synthase subunit beta, mitochondrial	ATPB_HUMAN	57	359
2	ATP synthase subunit alpha, mitochondrial	ATPA_HUMAN	60	260
3	4F2 cell-surface antigen heavy chain	4F2_HUMAN	68	198
4	**Sarcoplasmic/endoplasmic reticulum calcium ATPase 2**	AT2A2_HUMAN	115	148
5	**B-cell receptor-associated protein 31**	BAP31_HUMAN	28	120
6	**Cytoskeleton-associated protein 4**	CKAP4_HUMAN	66	108
7	Sodium/potassium-transporting ATPase subunit alpha	AT1A1_HUMAN	113	103
8	**Dolichyl-diphosphooligosaccharide--protein glycosyltransferase subunit 1**	RPN1_HUMAN	69	98
9	**Transmembrane emp24 domain-containing protein 10**	TMEDA_HUMAN	25	83
10	**Protein ERGIC-53**	LMAN1_HUMAN	58	69
11	**78 kDa glucose-regulated protein**	GRP78_HUMAN	72	64
12	Polypeptide N-acetylgalactosaminyltransferase 2	GALT2_HUMAN	65	63
13	**Dolichyl-diphosphooligosaccharide--protein glycosyltransferase 48 kDa subunit**	OST48_HUMAN	51	62
14	Transferrin receptor protein 1	TFR1_HUMAN	85	61
15	**Calnexin**	CALX_HUMAN	68	58
16	ATP synthase F(0) complex subunit B1, mitochondrial	AT5F1_HUMAN	29	58
17	ATP synthase subunit O, mitochondrial	ATPO_HUMAN	23	58
18	**Transmembrane emp24 domain-containing protein 9**	TMED9_HUMAN	27	53
19	ATP synthase subunit d, mitochondrial	ATP5H_HUMAN	18	53
20	**Inositol 1,4,5-trisphosphate receptor type 3**	ITPR3_HUMAN	304	52
21	**Vesicle-associated membrane protein-associated protein A**	VAPA_HUMAN	28	50
22	ATP synthase subunit gamma, mitochondrial	ATPG_HUMAN	33	49
23	Large neutral amino acids transporter small subunit 1	LAT1_HUMAN	55	47
24	Basigin	BASI_HUMAN	42	47
25	**Surfeit locus protein 4**	SURF4_HUMAN	30	46
26	**Inositol 1,4,5-trisphosphate receptor type 1**	ITPR1_HUMAN	314	44
27	**Very-long-chain enoyl-CoA reductase**	TECR_HUMAN	36	44
28	**Minor histocompatibility antigen H13**	HM13_HUMAN	41	44
29	Transmembrane protein 43	TMM43_HUMAN	45	42
30	Transmembrane 9 superfamily member 2	TM9S2_HUMAN	76	42
31	**Dolichyl-diphosphooligosaccharide-protein glycosyltransferase subunit 2**	RPN2_HUMAN	69	42
32	**Vesicle-trafficking protein SEC22b**	SC22B_HUMAN	25	41
33	Neutral amino acid transporter B(0)	AAAT_HUMAN	57	41
34	Monocarboxylate transporter 4	MOT4_HUMAN	49	40
35	Ubiquitin-60S ribosomal protein L40	RL40_HUMAN	15	38
36	CD44 antigen	CD44_HUMAN	82	37
37	Integrin beta-1	ITB1_HUMAN	88	37
38	**Transmembrane emp24 domain-containing protein 2**	TMED2_HUMAN	23	36
39	Polypeptide N-acetylgalactosaminyltransferase 1	GALT1_HUMAN	64	36
40	Phosphate carrier protein, mitochondrial	MPCP_HUMAN	40	34
41	Solute carrier family 35 member B1	S35B1_HUMAN	36	32
42	Transmembrane 9 superfamily member 3	TM9S3_HUMAN	68	31
43	**Translocon-associated protein subunit delta**	SSRD_HUMAN	19	31
44	**ER membrane protein complex subunit 1**	EMC1_HUMAN	112	29
45	**Transitional endoplasmic reticulum ATPase**	TERA_HUMAN	89	28
46	Golgi integral membrane protein 4	GOLI4_HUMAN	82	28
47	Podocalyxin	PODXL_HUMAN	59	28
48	**7-dehydrocholesterol reductase**	DHCR7_HUMAN	54	28
49	**Transmembrane emp24 domain-containing protein 7**	TMED7_HUMAN	25	28
50	**Vesicle-associated membrane protein-associated protein B/C**	VAPB_HUMAN	27	28
51	**Protein transport protein Sec61 subunit alpha isoform 1**	S61A1_HUMAN	52	27

Proteins were immunoprecipitated from mock-transfected, or SLC35B1-, or SLC35B1/Isoform 2-transfected HeLa cells with ANTI-FLAG M2 affinity gel and identified by mass spectrometry as previously described[18]. SLC35B1 and the 50 top hits are shown together with accession numbers, molecular weight (MW), and the number of peptides (Sum), which were detected in two parallel precipitates and corrected for the background in the negative control precipitate. We note that the expression level of Myc-DDK-tagged SLC35B1 in HeLa cells was very low when compared to a commercially available lysate from HEK293 cells, expressing the same Myc-DDK-tagged SLC35B1 (Supplementary Fig. 1). Proteins of the ER and ER-derived vesicles are given in bold face.

Supplementray Table 3 | Primers used for the generation of the *E.coli* expression constructs.

Primer	5´-sequence-3´	Construct
IsoI_fw	GGGGACAAGTTTGTACAAAAAAGCAGGCTTAATG GCCTCTAGCAGCTCC	Isoform 1 and Isoform 1 with C-terminal GFP
IsoII_fw	GGGGACAAGTTTGTACAAAAAAGCAGGCTTAATG AGGCCCCTGCCGCCG	Isoform 2
SCL35B1_rev	GGGGACCACTTTGTACAAGAAAGCTGGGTATTAG TGGGATGTCTTCTT	Isoforms 1 and 2
IsoI-GFP_rev	GGGGACCACTTTGTACAAGAAAGCTGGGTATTATT CTTCACCGGCATC	Isoform 1 with C-terminal GFP
