# Supplementary material for: AXER is an ATP/ADP exchanger in the membrane of the endoplasmic reticulum
Source: Nat Commun. 2018 Aug 28;9:3489. doi: 10.1038/s41467-018-06003-9 (PMC6113206; doi:10.1038/s41467-018-06003-9)
Supplement: Supplementary file 2 — Peer review_File [file 41467_2018_6003_MOESM2_ESM.pdf]

Reviewers' comments:

Reviewer #1 (Remarks to the Author):

The manuscript entitled "ACER is the ATP/ADP carrier in the ER membrane and part of a novel regulatory circuit" by Klein et al. deals with the important and long-awaited discovery of a transporter for ATP and ADP, named ACER, which is required to fuel the energy-requiring processes in the endoplasmic reticulum, such as protein folding. On the whole, this is an excellent paper and a good example of combined expertise being used to solve an important problem. The biggest problem is that the most important and experimentally supported discovery, i.e. ACER, is being diluted with some cell biology that is not well supported. I think it would be better to focus the paper solely on ACER, pulling in experimental evidence for it from the supplementary in the main part (unless it is a duplication) and to leave the more speculative parts on the regulation to a small part in the discussion, which could include Figure 4.
There are a few other points that require attention.
-The efflux experiments (Fig. 1 d) show that after the efflux reaches steady-state, the levels remain higher than starting value. Does this indicate that part of the ATP pool has been metabolized into a form that cannot be exchanged?
--The Extended Data Figure 5 | Ca^{2+}-CaM is a potential activator of SLC35B1 is not explained anywhere, but it is most likely to do with the proposed model of regulation in Figure 4.
-The docking suggested in the Extended Data Fig. 1b is highly unlikely, as it shows the adenine group, which is fairly hydrophobic, bound to a positively charged region, whereas the negative charged phosphate moieties are bound to neutral and hydrophobic residues. What is chemical basis of this pose? I am worried that the used docking program AutoDock4 might not consider waters or a variety of conformers. What other binding poses are there? Maybe it is good to show a few top-ranking poses. The problem with docking programs is that always give you a result, but you can never be sure it is correct. Here it is even more problematic as it deals with a model rather than an original structure.
-Line 625 "When SLC35B1 is modelled on the SnYddg transporter, two positive clusters and a potential ladder of aromatic residues suggest another analogy to the mitochondrial carriers, which involves the positive clusters in both steering the substrates to the central cavity and stripping Mg^{2+} from the nucleotides, respectively." In any case, this protein is not at all analogous to the mitochondrial ADP/ATP carriers as they have three pseudo-symmetrical domains and a completely different binding site. They only have three positively charged residues (not two different clusters). There is no discernable aromatic ladder. If a comparison is made why is it not referenced? Why not show the comparison?
Minor corrections
-The paper is not structured as a Nat. Comms paper.
-line 78 was effectively facilitating the uptake of radiolabeled ATP or ADP into the cells > facilitated the uptake of radiolabeled ATP or ADP into the cells effectively.
-line 93 an energy transporter > transporter of chemical energy
-line 100 etc "However, the Western blot approach failed in the absence of SLC35B1 over-expression, probably due to the low level of native SLC35B1 in HeLa cells (15 nM; Extended Data Table 2)" Not sure what this line means.
-The loading in the lanes of Fig. 1a are not described in the legend nor are the different constructs shown in the figure explained in the text.
Line 530 we considered the open Phyre2 as more promising for a ligand docking study > we considered the open Phyre2 to be more promising for a ligand docking study.

Reviewer #2 (Remarks to the Author):

9-Feb-18

ATP is a substrate in various important reactions that take place in the lumen of the ER, most notably the BiP/Hsp70 chaperone cycle. Yet the means by which lumenal pools of ATP are maintained has remained a mystery. The authors took a bioinformatics approach to identify candidate genes that might encode an ER-localized transmembrane ATP transporter. This effort led them to SLC35B1 (renamed here ACER), a member of the large family of solute carriers, whose expression pattern correlates with BiP. This is a reasonable criterion to begin the search, but its specificity is undermined by the fact that BiP is a housekeeping protein, whose level varies between cells largely on the basis of the size of their ER. Thus the bioinformatics effort, as executed here can, at most, provide a subtle clue as to the identity of the transporter; the indictment of SLC35B1 must rest on experimental data and the review of this paper is therefore an attempt to assess the strength of that experimental evidence.

The claim that SLC35B1 is an ER localized protein rests on the ER-localization of an over-expressed GFP tagged version of the protein. This is a weak criterion, as ER retention of any over-expressed SLC, even one that is normally destined for another membrane compartment, would look very much the same. Unfortunately, the authors are unable to detect the endogenous protein with available antisera, which could well reflect weakness of the immunochemicals. This weakness might be traversed by knocking-in a tag into the endogenous SLC35B1, using CRISPR/Cas9 technology – not an unreasonable expectation of a paper in 2018.

The biophysical data supporting the claim that SLC35B1 exchanges ATP for ADP is rests largely on evidence that E. coli expressing SLC35B1 accumulate 32P when incubated in the presence of ATP radiolabeled on its alpha phosphate (Figure 1B & 1C). This is a reasonable first step, but falls far short of what might be required to convince a sceptic that SLC35B1 is an exchanger. What is the evidence that an exchange with ADP underlies the transport? What is the evidence that the radiolabeled species accumulating in the bacterial cells is ATP? The paper makes mention of the saturable kinetics of ATP/ADP transport ("Heterologously expressed SLC35B1 exhibited similar apparent KM and Vmax values 83 for ATP (33.3 ± 2.0 μM and 881.7 ± 119.8 pmol/mg protein and h) and ADP (32.8 ± 7.4 μM 84 and 827.3 ± 77.0 pmol/mg protein and h"), but it unclear where the primary data supporting this claim might be found.

It is notoriously difficult to express functional multi-pass eukaryotic transmembrane proteins in bacteria, yet the authors claim to have done so successfully in this case. What, beyond the transport assay shown in figure 1B & 1C, is the evidence that bacterially-expressed SLC35B1 is correctly integrated into the membrane? Have the authors correlated mutations that are (predicted) to disrupt function with loss of transport in the E. coli-based assay?

To support their claim that the transport kinetics of bacterially-expressed SLC35B1 match those of an endogenous ER-localised ATP transporter, the authors refer to REF 23 (Mayinger, P., Bankaitis, V. A. & Meyer, D. I. Sac1p mediates the adenosine triphosphate transport into yeast endoplasmic reticulum that is required for protein translocation. J. Cell Biol. 131, 1377-1386, 1995, PMID: 8522598). The conclusions of that paper (that Sac1p is an ER-localized yeast ATP transporter) are rather controversial, but even if, as the authors suggest, REF 23 is to be credited with having measured kinetics of ATP transport into the ER, then it should be possible for the authors to conduct a related set of measurements by exploiting the methods of Mayinger et al. 1995 and eukaryotic expression of SLC35B1, which is much more likely to yield a correctly folded protein.

The cell based work reported on in figures 2 and 3 follows a rather convoluted logic whereby the loss of SLC35B1-mediated transport of ATP is reflected in lowered ATP concentration in the ER which selectively affects BiP's ability to serve as plug preventing a calcium leak via the Sec61 nascent peptide transporter. Leaving aside the question of whether or not BiP has such a role in ER calcium metabolism (a matter that remains unresolved, in the opinion of many cell biologists), the question remains as to why the loss of ATP would affect this process and not other features of BiP function, such as its ATP-dependent chaperone function, which would be reflected in activation of unfolded protein stress pathways. That a UPR is not induced in SLC35B1 knockdown cells is not an argument against it being a/the ATP transporter (it is merely a weak experiment with limited interpretability – as residual levels of ATP in the ER may be high enough to sustain BiP function), but it seems rather unlikely that the lower ATP concentrations, brought about by the SLC35B1

knockdown selectively compromise one aspect of BiP function, over another.

Response to the comments of the referees:

We note that the manuscript was originally submitted to another Nature journal and directly transferred to Nature Communications upon our request. We are convinced that the space limitations of this other journal may have caused at least some of the objections of the reviewers. In retrospect, it appears to be our mistake to refrain from re-formating our manuscript in the course of journal transfer.

Reviewer #1

Reviewer #1: I think it would be better to focus the paper solely on ACER, pulling in experimental evidence for it from the supplementary in the main part (unless it is a duplication) and to leave the more speculative parts on the regulation to a small part in the discussion, which could include Figure 4.

We have followed your advice and shifted the focus of the manuscript to ACER by both further characterizing various heterologously expressed SLC35B1 variants and comparing their transport characteristics to ATP import into proteoliposomes, harboring the full complement of mammalian ER membrane proteins. These data are shown in new Figs. 2-4 plus Table 1 plus Supplementary Figs. 2-5 and discussed in the Results on pages 5-7.

Furthermore, we discuss the impact of SLC35B1 depletion from HeLa cells on ER protein import and cellular calcium homeostasis as additional albeit indirect evidence for the fact that SLC35B1 depletion reduces BiP activity, as a secondary effect of ATP depletion. This is shown in new Fig. 8 and described in the Results on pages 9 and 10.

Reviewer #1: There are a few other points that require attention.
-The efflux experiments (Fig. 1 d) show that after the efflux reaches steady-state, the levels remain higher than starting value. Does this indicate that part of the ATP pool has been metabolized into a form that cannot be exchanged?

Indeed, because of the metabolic activity of *E. coli* cells some [^{32}P]ATP is converted into labeled ADP, AMP, and inorganic phosphate (P_i). We have addressed this by thin layer chromatography of the supernatant from a similar experiment and show the result in new Supplementary Fig. 2. It supports the conclusion that SLC35B1 acts as antiporter.

--The Extended Data Figure 5 | Ca2+-CaM is a potential activator of SLC35B1 is not explained anywhere, but it is most likely to do with the proposed model of regulation in Figure 4.

We have added the missing explanation to the Discussion on page 13.

-The docking suggested in the Extended Data Fig. 1b is highly unlikely, as it shows the adenine group, which is fairly hydrophobic, bound to a positively charged region, whereas the negative charged phosphate moieties are bound to neutral and hydrophobic residues. What is chemical basis of this pose? I am worried that the used docking program AutoDock4 might not consider waters or a variety of conformers. What other binding poses are there? Maybe it is good to show a few top-ranking poses. The problem with docking programs is that always give you a result, but you can never be sure it is correct. Here it is even more problematic as it deals with a model rather than an original structure.

We agree that docking results obtained by protein-ligand docking need to be interpreted with caution. Also, we agree that docking into homolgy models is more problematic than docking into an X-ray structure as was correctly pointed out. The point of the docking study was not to show that ATP binds to SLC35B1. This is clearly shown by experiment. The docking study was initiated to suggest potential binding modes of ATP inside the carrier. As will be pointed out, the criticism of the reviewer about an incorrect „chemistry“ of the suggested docking pose is not justified in our view. However, we agree that Extended Data Fig. 1b gave a misleading impression of the outcome of the docking analysis. We have followed the advice of the reviewer and prepared a novel Supplementary Fig. 7, which shows the six top-ranked poses for ATP binding to SLC35B1. These results are discussed in the Discussion on pages 12 and 13. In these poses, the negative phosphate groups are coordinated by the same set of positively charged amino acid residues (Lys 117, Lys 120, Arg 276), the H-bonding atoms of the adenine ring are coordinated by polar groups, and the adenine ring is positioned close to aromatic residues (Tyr 28, Tyr 94). Such contacts are typical coordination scenarios of ATP bound to proteins. Hence, all six docking poses can be considered as plausible binding modes.

-Line 625 "When SLC35B1 is modelled on the SnYddg transporter, two positive clusters and a potential ladder of aromatic residues suggest another analogy to the mitochondrial carriers, which involves the

positive clusters in both steering the substrates to the central cavity and stripping Mg2+ from the nucleotides, respectively." In any case, this protein is not at all analogous to the mitochondrial ADP/ATP carriers as they have three pseudo-symmetrical domains and a completely different binding site. They only have three positively charged residues (not two different clusters). There is no discernable aromatic ladder. If a comparison is made why is it not referenced? Why not show the comparison?

We have moved this section to the Discussion on pages 12 and 13 and extended it to: When SLC35B1 is modelled on the X-ray structure of the *Sn*Yddg transporter, two positive clusters that are flanking transmembrane domain 5 on the cytosolic and ER lumenal sides (Fig. 1a, b, Supplementary Fig. 7) suggest another analogy to the mitochondrial carriers, whereby the positive clusters are involved in both steering the substrates to the central cavity and stripping Mg^{2+} from the nucleotides, respectively. Notably, a stripping of Mg^{2+} from the nucleotides is consistent with the observed EDTA insensitivity of both heterologously expressed SLC35B1 and the carrier, which is present in proteoliposomes, harboring the full complement of mammalian ER membrane proteins (Table 1, Fig. 4b). In further analogy to the mitochondrial carriers, transport direction can be expected to be directed by the ATP gradient.

We omitted the reference to the ladder of aromatic residues, since there is another SLC35B1 variant, transcript variant 2, which lacks it and yet has some residual carrier activity (Supplementary Figure 3a, b).

Reviewer #1: Minor corrections
-The paper is not structured as a Nat. Comms paper.
The manuscript was originally submitted to another Nature journal and directly transferred.
-line 78 was effectively facilitating the uptake of radiolabeled ATP or ADP into the cells > facilitated the uptake of radiolabeled ATP or ADP into the cells effectively.
Corrected as suggested.
-line 93 an energy transporter > transporter of chemical energy
Corrected as suggested.
-line 100 etc "However, the Western blot approach failed in the absence of SLC35B1 over-expression, probably due to the low level of native SLC35B1 in HeLa cells (15 nM; Extended Data Table 2)" Not sure what this line means.
Changed to: However, the Western blot approach for endogeneous SLC35B1 failed, most likely due to the low level of native SLC35B1 in HeLa cells[17] (see Supplementary Fig. 1e).
We refer to the SLC35B1 concentration in HeLa cells and the related Supplementary Table 1 in the Results on page 4.
-The loading in the lanes of Fig. 1a are not described in the legend nor are the different constructs shown in the figure explained in the text.
Changed to: The indicated SLC35B1 variants (labeled by asterisk in **a**), which were described in the legend to Fig. 1a, plus a phosphomimetic variant of Isoform 2 with aspartate residues in positions 15 and 29 instead of serines (termed Isoform 2DD, lane 5), and the GFP-tagged versions of SLC35B1 were expressed in different *E. coli* cells. (**a, b**) Membranes were isolated from the indicated *E. coli* cells, and samples (25 µg protein) analysed by SDS-PAGE and Western blotting using an SLC35B1-specific antibody, which was characterized in Supplementary Fig 1a (**a**). Non-induced cells served as a negative control and are shown in lane 1. Coomassie staining the blot for total protein served as a loading control (**b**).
Line 530 we considered the open Phyre2 as more promising for a ligand docking study > we considered the open Phyre2 to be more promising for a ligand docking study.
Corrected as suggested.

Reviewer #2
Reviewer #2: The authors took a bioinformatics approach to identify candidate genes that might encode an ER-localized transmembrane ATP transporter. This effort led them to SLC35B1 (renamed here ACER), a member of the large family of solute carriers, whose expression pattern correlates with BiP. This is a reasonable criterion to begin the search, but its specificity is undermined by the fact that BiP is a housekeeping protein, whose level varies between cells largely on the basis of the size of their ER. Thus the bioinformatics effort, as executed here can, at most, provide a subtle clue as to the identity of the transporter; the indictment of SLC35B1 must rest on experimental data and the review of this paper is therefore an attempt to assess the strength of that experimental evidence.

It may have been misleading to introduce SLC35B1 as an ATP carrier candidate in the way we did, but the manuscript is not about the bioinformatics strategy of how we got to the protein. Fact is, we found the carrier as described and got lucky. We can easily omit this if necessary.

Independently of the exact way of identifying SLC35B1, we followed your advice with respect to experimental evidence and shifted the focus of the manuscript to ACER by both further characterizing various heterologously expressed SLC35B1 variants and comparing their transport characteristics to ATP import into proteoliposomes, harboring the full complement of mammalian ER membrane proteins. We found that these characteristics (antiport mode, K_M, substrate specificity, EDTA insensitivity) correlate very well, further supporting the conclusion that SLC35B1 is the elusive ATP/ADP carrier of the ER membrane. These new data are shown in new Figs. 2-4 plus Supplementary Figs. 2-5 and discussed on pages 5-7.

Reviewer #2: The claim that SLC35B1 is an ER localized protein rests on the ER-localization of an over-expressed GFP tagged version of the protein. This is a weak criterion, as ER retention of any over-expressed SLC, even one that is normally destined for another membrane compartment, would look very much the same. Unfortunately, the authors are unable to detect the endogenous protein with available antisera, which could well reflect weakness of the immunochemicals. This weakness might be traversed by knocking-in a tag into the endogenous SLC35B1, using CRISPR/Cas9 technology – not an unreasonable expectation of a paper in 2018.

As described by Fumagalli et al., Nat. Cell Biol. 18, 1173-84, we successfully manged to implement the CRISPR/Cas9 technology in HEK 293 cells. However, we and others have failed to get CRISPR/Cas9 to work in HeLa cells, most likely due to their polyploidy.

However, we went to great lengths to demonstrate that SLC35B1 is an ER membrane protein in mammalian cells. 1) By subjecting the equivalent of 6 mg of rough microsomal protein from canine pancreas to SDS-PAGE and Western blot, we were able to detect the protein, strongly suggesting that SLC35B1 is a true ER membrane protein. Typically, 25 µg of microsomal protein are sufficient to detect your average protein of interest in these microsomes. We also subjected 500 µg of HeLa cell protein to SDS-PAGE and Western blot but failed to detect SLC35B1 (Supplementary Fig. 1). This is consistent with the low SLC35B1 level, which was determined by Matthias Mann´s lab for HeLa cells (Hein et al., Cell 163, 712-723). We estimate that we would have to subject 12 mg of HeLa cell protein to SDS-PAGE, which is not feasible. 2) We aimed to use low expression levels of plasmid encoded SLC35B1 in our experiments. To confirm that we have succeeded to work with low expression levels, we have added a new Supplementary Fig. 1 to show this for the GFP- as well as the Myc-DDK-tagged variant. Therefore, we kept the images of GFP-tagged SLC35B1 in Fig. 1d as one piece of evidence for the ER localization of SLC35B1 in intact human cells. Furthermore, we performed immunoprecipitations of the Myc-DDK-tagged version of the protein and got mass spectrometry results for the precipitate, which are typical for an ER resident protein. These new results are given in the first section of the Results on pages 4 and 5 and Supplementary Table 2.

Reviewer #2: The biophysical data supporting the claim that SLC35B1 exchanges ATP for ADP is rests largely on evidence that E. coli expressing SLC35B1 accumulate 32P when incubated in the presence of ATP radiolabeled on its alpha phosphate (Figure 1B & 1C). This is a reasonable first step, but falls far short of what might be required to convince a sceptic that SLC35B1 is an exchanger. What is the evidence that an exchange with ADP underlies the transport? What is the evidence that the radiolabeled species accumulating in the bacterial cells is ATP? The paper makes mention of the saturable kinetics of ATP/ADP transport ("Heterologously expressed SLC35B1 exhibited similar apparent KM and Vmax values 83 for ATP (33.3 ± 2.0 µM and 881.7 ± 119.8 pmol/mg protein and h) and ADP (32.8 ± 7.4 µM 84 and 827.3 ± 77.0 pmol/mg protein and h"), but it unclear where the primary data supporting this claim might be found.

The first evidence that heterologously expressed SLC35B1 is an exchanger is presented in new Figs. 3a, b plus Supplementary Fig. 2 and shows that newly imported radiolabeled ATP can indeed be chased out of the cells by an excess of unlabelled ATP. Secondly, we prepared proteoliposomes from the membranes of transfected *E. coli* cells and observed reconstituted SLC35B1 to be able to facilitate the import of ATP or ADP into these proteoliposomes and to operate in antiport mode, i.e. nucleotide import into the proteoliposomes was dependent on their preloading with ADP or ATP (Fig. 3c-f). We have also added the missing primary data for K_M and V_{max} in new Supplementary Fig. 4.

In addition, we both further charcterized heterologously expressed SLC35B1 and compared its transport characteristics to ATP import into proteoliposomes, harboring the full complement of mammalian ER membrane proteins. As we report in the Results on pages 6 and 7 and new Fig. 4 plus Supplementary Fig. 5, substrate specificity and antiport mode of SLC35B1 were also shared by the ATP carrier, which was present in proteoliposomes, harboring the full

complement of mammalian ER membrane proteins. In this case, the antiport mode was deduced from the fact that ATP import into proteoliposomes was dependent on their preloading with ADP.

Reviewer #2: It is notoriously difficult to express functional multi-pass eukaryotic transmembrane proteins in bacteria, yet the authors claim to have done so successfully in this case. What, beyond the transport assay shown in figure 1B & 1C, is the evidence that bacterially-expressed SLC35B1 is correctly integrated into the membrane? Have the authors correlated mutations that are (predicted) to disrupt function with loss of transport in the E. coli-based assay?

We followed the reviewer´s advice and analyzed a mutant (it is listed as a splice variant) which lacks the first two transmembrane domains plus a cluster of aromatic reidues. This mutant is expected to display a reduced functionality. As we report in the Results on page 6 and new Supplementary Fig. 3a, b this significantly shortened variant of SLC35B1 showed much lower transport rates for ATP and ADP as compared to Isoforms 1 and 2, although it is present in the *E. coli* membranes at similar concentrations as the other two isoforms (Fig. 2a). Together with the new functional data (see below), these new results confirm that we succeeded to integrate the functional human proteins into the bacterial membranes. We also note that one co-author of the manuscript, Ekkehard Neuhaus has built almost his entire career on the successful use of this approach (eg Jung et al., Nat. Plants, 14001; Leroch et al., Plant Cell 20, 438-451; full list on https://www.bio.uni-kl.de/pflanzenphysiologie/publikationen/).

Furthermore, we prepared proteoliposomes from the membranes of transfected *E. coli* cells and observed reconstituted SLC35B1 to be able to facilitate the import of ATP or ADP into these proteoliposomes and to operate in antiport mode, i.e. nucleotide import into the proteoliposomes was dependent on their preloading with ADP or ATP (Fig. 3c-f). Thus heterologously expressed SLC35B1 is a native carrier protein in bacterial plasma membranes.

Reviewer #2: To support their claim that the transport kinetics of bacterially-expressed SLC35B1 match those of an endogenous ER-localised ATP transporter, the authors refer to REF 23 (Mayinger, P., Bankaitis, V. A. & Meyer, D. I. Sac1p mediates the adenosine triphosphate transport into yeast endoplasmic reticulum that is required for protein translocation. J. Cell Biol. 131, 1377-1386, 1995, PMID: 8522598). The conclusions of that paper (that Sac1p is an ER-localized yeast ATP transporter) are rather controversial, but even if, as the authors suggest, REF 23 is to be credited with having measured kinetics of ATP transport into the ER, then it should be possible for the authors to conduct a related set of measurements by exploiting the methods of Mayinger et al. 1995 and eukaryotic expression of SLC35B1, which is much more likely to yield a correctly folded protein.

We did not want to give the impression that we believe Sac1p is an ATP carrier, on the contrary. We just used this reference for comparison of our kinetics with the kinetics of the ATP carrier, which is present in yeast ER membranes. We have added a sentence to the Results on page 7 to settle this issue.

Furthermore, as you suggested, we compared the transport characteristics of heterologously expressed SLC35B1 to ATP import into proteoliposomes, harboring the full complement of mammalian ER membrane proteins (see above). As we report in the Results on pages 6 and 7 and new Fig. 4 plus Supplementary Fig. 5, substrate specificity and antiport mode of SLC35B1 are also shared by the ATP carrier, which was present in proteoliposomes, harboring the full complement of mammalian ER membrane proteins. In this case, the antiport mode was deduced from the fact that ATP import into proteoliposomes was dependent on their preloading with ADP.

Reviewer #2: The cell based work reported on in figures 2 and 3 follows a rather convoluted logic whereby the loss of SLC35B1-mediated transport of ATP is reflected in lowered ATP concentration in the ER which selectively affects BiP's ability to serve as plug preventing a calcium leak via the Sec61 nascent peptide transporter. Leaving aside the question of whether or not BiP has such a role in ER calcium metabolism (a matter that remains unresolved, in the opinion of many cell biologists), the question remains as to why the loss of ATP would affect this process and not other features of BiP function, such as its ATP-dependent chaperone function, which would be reflected in activation of unfolded protein stress pathways. That a UPR is not induced in SLC35B1 knockdown cells is not an argument against it being a/the ATP transporter (it is merely a weak experiment with limited interpretability – as residual levels of ATP in the ER may be high enough to sustain BiP function), but it seems rather unlikely that the lower ATP concentrations, brought about by the SLC35B1 knockdown selectively compromise one aspect of BiP function, over another.

We did not want to give the impression that there is any selectivity of ATP depletion on BiP functions. Indeed, one can expect protein folding to be negatively affected by SLC35B1 depletion. The point is that we do our cell based assays at a time point where protein misfolding apparently is not yet severe enough to trigger UPR (new Fig. 5a, b), yet it is sufficient to lower the ER ATP content and affect BiP functions, such as gating of the Sec61 channel to the open state for protein import and to the closed state for maintaining the steep ER to cytosol calcium gradient. In fact, we show in new Fig. 8a-d that BiP´s function in protein import is negatively affected by ATP depletion. Furthermore, we discuss the impact of SLC35B1 depletion from HeLa cells on cellular calcium homeostasis (new Fig. 8e-l) as additional, albeit indirect evidence for the fact that it reduces BiP activity, thereby, shifting the focus of the entire section of the manuscript to the ATP carrier function of SLC35B1.

However, we also kept our initial experiments in the manuscript, which address a possible mechanism for regulation of ATP transport into the ER since we are convinced that the manuscript benefits from this novel concept for a calcium dependent regulatory circuit, which guarantees sufficient ATP supply to the ER under normal and stress conditions (new Figure 8m, n).

Reviewer #1 (Remarks to the Author):

The manuscript is much improved and now focusses largely on the discovery of ACER, which will have a major impact on the cell biology of the ER.

Minor corrections of the revised version

It is of course the prerogative of the discoverers to name the protein, but I wonder whether it would not be better to avoid the term ADP/ATP carrier because of the direct association with the mitochondrial ADP/ATP carrier, which belongs to a completely different protein family, which is confusing. The authors use different terms throughout; transporter, carrier, importer/exporter but what about "exchanger", which also reflects the transport function better. Of course, this would mean that the name needs to be changed, e.g. AXER

Line 71 ten transmembrane domains > ten transmembrane helices

Line 289 which is present > that is present

Line 296 agorose > agarose

Line 495 atoministic structure > comparative atomic model

Supplementary Figure 5 The problem with the Eadie–Hofstee approach is that neither ordinate or abscissa represent independent variables, as both are dependent on the reaction rate. Thus, any experimental error will propagate unevenly and become larger over the abscissa, thereby giving more weight to smaller values of v/[S]. Better to use iteration (curve fitting) to determine the kinetic parameters.

Reviewer #2 (Remarks to the Author):

The revised manuscript contains a wealth of new information.

Of particular note are experiments that measure the kinetic properties of the transport in preparations of liposomes reconstituted with mammalian ER components (Figure S5). These serve as a reference for the kinetics observed in whole cells (E. coli) expressing the mammalian protein (Figure S4). In a separate place (Figure 3) the rate of import of hot ATP into proteoliposomes constituted with membrane proteins derived from E. coli expressing SLC35B1 is shown to be dependent on the proteoliposomes having been pre-loaded with ADP (an important criteria to convince the reader that an exchange mechanism is at play).

Other new experiments (Figure S2), evaluate the efflux of the products derived from the hot ATP that made its way into the SLC35B1-expressing E. coli. However this experiment seems marred by the absence of a control in which the same procedure is carried out on E. coli that do not express SLC35B1.

While important improvements have been made to the manuscript, the end product is not a paper that would convince a critical reader that the authors have discovered a protein that ferries ATP into the mammalian ER. It is as though the authors do not wish to strongly make that point – the most convincing bits of data are relegated to disparate

supplemental figures and the manuscript remains heavily laden with irrelevant observations. This seems to have come about from the attempt to convert an earlier version of the story, destined for a different venue, to one before us.

This reviewer would be convinced by a manuscript with two key elements displayed clearly (not peppered through an inchoate manuscript):

A. SLC35B1 is an ER localised protein. If the antisera cannot detect it – I suggested knocking in a tag to the endogenous locus. The authors rebuttal to this suggestion - that the polyploidy of Hela cells stands in the way - is difficult to accept as the suggestion is not that they eliminate gene function by editing, but rather that they introduce a tag to one/few/all of the gene copies in the cells. Given the functionality of the GFP-tagged SLC35B1 (figure S3e and S3f), they already know where to place the tag, while preserving function.

B. A side by side comparison showing that proteoliposomes constituted with E. coli expressed SLC35B1 (such as those used in the experiment shown in figure 3) have acquired the same transport features as proteoliposomes constituted with mammalian ER proteins (such as those used in figure S5). This comparison would be made all the more convincing if the authors could also render liposomes constituted with membrane proteins from mammalian cells over-expressing SLC35B1 (I should think this would be easier to produce than the E. coli derived samples and more believable too)

And a final note: At the author's discretion, Nature communications provides an option to have the reviewer's comments published alongside the manuscript. If this manuscript is published in its current form, it is my fervent hope that the authors will have the courage to select this option. They should do so if they believe their conclusion: if nothing else to show the world that they were right and the pedantic reviewer #2 was wrong! And if it turns out, as I fear it will, that SLC35B1 (like Sac1p of yore) has nothing to do with ATP transport into the ER, give me the satisfaction of seeing this highly critical review (and the one of the earlier version of the paper) in print.

Reviewer #1 (Remarks to the Author):

The manuscript is much improved and now focusses largely on the discovery of ACER, which will have a major impact on the cell biology of the ER.

Minor corrections of the revised version

It is of course the prerogative of the discoverers to name the protein, but I wonder whether it would not be better to avoid the term ADP/ATP carrier because of the direct association with the mitochondrial ADP/ATP carrier, which belongs to a completely different protein family, which is confusing. The authors use different terms throughout; transporter, carrier, importer/exporter but what about “exchanger”, which also reflects the transport function better. Of course, this would mean that the name needs to be changed, e.g. AXER
We followed your advice.

Line 71 ten transmembrane domains > ten transmembrane helices
We changed the wording as requested.

Line 289 which is present > that is present
We changed the wording as requested.

Line 296 agorose > agarose
We corrected the spelling.

Line 495 atoministic structure > comparative atomic model
We changed the wording as requested.

Supplementary Figure 5 The problem with the Eadie–Hofstee approach is that neither ordinate or abscissa represent independent variables, as both are dependent on the reaction rate. Thus, any experimental error will propagate unevenly and become larger over the abscissa, thereby giving more weight to smaller values of v/[S]. Better to use iteration (curve fitting) to determine the kinetic parameters.
We followed your advice. To determine V_{max} (maximal velocity) and K_m (Michaelis constant) the measured values were fitted to the Hill equation $y = V_{max} \times x^n/(k^n + x^n)$ with $n = 1$ for noncooperative binding. Calculations were performed with Origin 8 (OriginLab Corp.).

Reviewer #2 (Remarks to the Author):

The revised manuscript contains a wealth of new information.

Of particular note are experiments that measure the kinetic properties of the transport in preparations of liposomes reconstituted with mammalian ER components (Figure S5). These serve as a reference for the kinetics observed in whole cells (E. coli) expressing the mammalian protein (Figure S4). In a separate place (Figure 3) the rate of import of hot ATP into proteoliposomes constituted with membrane proteins derived from E. coli expressing SLC35B1 is shown to be dependent on the proteoliposomes having been pre-

loaded with ADP (an important criteria to convince the reader that an exchange mechanism is at play).

Other new experiments (Figure S2), evaluate the efflux of the products derived from the hot ATP that made its way into the SLC35B1-expressing E. coli. However this experiment seems marred by the absence of a control in which the same procedure is carried out on E. coli that do not express SLC35B1.

While important improvements have been made to the manuscript, the end product is not a paper that would convince a critical reader that the authors have discovered a protein that ferries ATP into the mammalian ER. It is as though the authors do not wish to strongly make that point – the most convincing bits of data are relegated to disparate supplemental figures and the manuscript remains heavily laden with irrelevant observations. This seems to have come about from the attempt to convert an earlier version of the story, destined for a different venue, to one before us.

This reviewer would be convinced by a manuscript with two key elements displayed clearly (not peppered through an inchoate manuscript):

A. SLC35B1 is an ER localised protein. If the antisera cannot detect it – I suggested knocking in a tag to the endogenous locus. The authors rebuttal to this suggestion - that the polyploidy of Hela cells stands in the way - is difficult to accept as the suggestion is not that they eliminate gene function by editing, but rather that they introduce a tag to one/few/all of the gene copies in the cells. Given the functionality of the GFP-tagged SLC35B1 (figure S3e and S3f), they already know where to place the tag, while preserving function.
We agree that it is always better to have a second and even a third confirmation regarding any scientific question. In this case, this would mean a second demonstration of tagged SLC35B1 in the ER of HeLa cells (in addition to the GFP tagged variant in Figure 1d). However, we don´t see how a stable cell line could simultaneously provide enough protein for fluorescence microscopy to work and yet not too much overproduction, as you correctly pointed out as a potential problem in your first review. We are convinced that the used transient transfection gave us a better control for expression levels as can be expected from the suggested CRISPR/cas9 approach. We refer to this point in a caveat in lines 120-129, as requested by the editor.

B. A side by side comparison showing that proteoliposomes constituted with E. coli expressed SLC35B1 (such as those used in the experiment shown in figure 3) have acquired the same transport features as proteoliposomes constituted with mammalian ER proteins (such as those used in figure S5). This comparison would be made all the more convincing if the authors could also render liposomes constituted with membrane proteins from mammalian cells over-expressing SLC35B1 (I should think this would be easier to produce than the E. coli derived samples and more believable too)
The requested comparison between proteoliposomes with *E. coli* expressed SLC35B1 and proteoliposomes constituted with mammalian ER proteins was shown in Figures 3c-f and 4a. We agree that it would be nice to also have proteoliposomes with HeLa expressed SLC35B1 for futher comparison, but are convinced that it is not technically feasilbe to purify ER from HeLa cells in the required quantity and purity (i.e. without contaminating mitochondria). We refer to this point in a caveat in lines 199-204, as requested by the editor.

And a final note: At the author's discretion, Nature communications provides an option to have the reviewer's comments published alongside the manuscript. If this manuscript is published in its current form, it is my fervent hope that the authors will have the courage to select this option. They should do so if they believe their conclusion: if nothing else to show the world that they were right and the pedantic reviewer #2 was wrong! And if it turns out, as I fear it will, that SLC35B1 (like Sac1p of yore) has nothing to do with ATP transport into the ER, give me the satisfaction of seeing this highly critical review (and the one of the earlier version of the paper) in print.
We were made aware of this option by the editor and would have selected it anyway. We note that Nature communications provides reviewers the option to have their names published in the peer review files and would appreciate if reviewer #2 would select this option.

Yours sincerely,
